# Heteroatom-Induced Accelerated Kinetics on Nickel Selenide for Highly Efficient Hydrazine-Assisted Water Splitting and Zn-Hydrazine Battery

**DOI:** 10.1007/s40820-023-01128-z

**Published:** 2023-06-19

**Authors:** Hao-Yu Wang, Lei Wang, Jin-Tao Ren, Wen-Wen Tian, Ming-Lei Sun, Zhong-Yong Yuan

**Affiliations:** 1 School of Materials Science and Engineering, Smart Sensing Interdisciplinary Science Center, Nankai University, Tianjin, 300350 People’s Republic of China; 2 Key Laboratory of Advanced Energy Materials Chemistry (Ministry of Education), Nankai University, Tianjin, 300071 People’s Republic of China

**Keywords:** Water electrolysis, Hydrogen production, Hydrazine oxidation, Bifunctional electrocatalyst, Heteroatom doping

## Abstract

Hydrazine-assisted water electrolysis is a promising energy conversion technology for highly efficient hydrogen production. Rational design of bifunctional electrocatalysts, which can simultaneously accelerate hydrogen evolution reaction (HER)/hydrazine oxidation reaction (HzOR) kinetics, is the key step. Herein, we demonstrate the development of ultrathin P/Fe co-doped NiSe_2_ nanosheets supported on modified Ni foam (P/Fe-NiSe_2_) synthesized through a facile electrodeposition process and subsequent heat treatment. Based on electrochemical measurements, characterizations, and density functional theory calculations, a favorable “2 + 2” reaction mechanism with a two-step HER process and a two-step HzOR step was fully proved and the specific effect of P doping on HzOR kinetics was investigated. P/Fe-NiSe_2_ thus yields an impressive electrocatalytic performance, delivering a high current density of 100 mA cm^−2^ with potentials of − 168 and 200 mV for HER and HzOR, respectively. Additionally, P/Fe-NiSe_2_ can work efficiently for hydrazine-assisted water electrolysis and Zn-Hydrazine (Zn-Hz) battery, making it promising for practical application.

**Supplementary Information:**

The online version contains supplementary material available at 10.1007/s40820-023-01128-z.

## Highlights

- Ultrathin P and Fe co-doped NiSe_2_ nanosheets supported on modified Ni foam are synthesized, which shows desirable bifunctional electrocatalytic hydrogen evolution reaction (HER)/hydrazine oxidation reaction (HzOR) performance in hydrazine-assisted water electrolysis and Zn-Hz battery.
- The coexistence of P and Fe heteroatoms induces an accelerated “2 + 2” reaction mechanism with a two-step HER process and a two-step HzOR step.

**Supplementary Information:**

The online version contains supplementary material available at 10.1007/s40820-023-01128-z.

## Graphical abstract

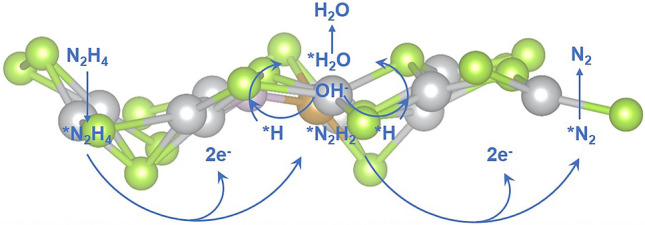

## Introduction

Among various substitutes for fossil fuels, hydrogen, a green, clean and renewable energy resource with high gravimetric energy density, is considered as one of the most promising alternatives [1, 2]. Electrocatalytic water splitting is a promising pathway for the production of high-purity hydrogen with zero carbon emission [3–8]. Since oxygen evolution reaction (OER) with high thermodynamic oxidation potential and complex four-electron transfer process is unbefitting and obstructive in practical application, various small molecules can participate into the oxidation process at the anode to replace sluggish OER process, such as hydrazine, ammonia, urea, methanol and ethanol [9–11]. Among them, hydrazine oxidation reaction (HzOR) with a high theoretical working potential advantage of 1.56 V compared with OER (OER: 1.23 V *vs.* RHE; HzOR: − 0.33 V *vs.* RHE) can be a promising alternative [12]. Integrating cathodic hydrogen evolution reaction (HER) process and anodic HzOR process to construct a hydrazine-assisted water electrolyzer can efficiently decrease the applied cell voltage and avoid the mixing of anodic O_2_ and cathodic H_2_ in membrane-free devices [13].

To simultaneously accelerate the reaction kinetics of both HER and HzOR, several properties should be given to electrocatalysts. In terms of HER process, non-noble metal electrocatalysts generally catalyze a Volmer–Heyrovsky mechanism in alkaline media [14–16]:

1 $$ {\text{Volmer step}}:{\text{H}}_{2} {\text{O }} + {\text{ M }} + {\text{ e}}^{ - }  \to {\text{M}} - {\text{H}}_{{{\text{ad}}}}  + {\text{ OH}}^{ - }$$

2 $$ {\text{Heyrovsky step}}:{\text{ M}} - {\text{H}}_{{{\text{ad}}}} + {\text{ H}}_{2} {\text{O }} + {\text{ e}}^{ - } \to {\text{ M }} + {\text{ H}}_{2} + {\text{ OH}}^{ - } $$

Herein, M and H_ad_ are the surface active sites and the absorbed hydrogen, respectively. The sluggish dissociation process of water molecule and adsorption of H in Volmer step makes it the rate-determining step (RDS) in HER process. Consequently, efficient HER electrocatalyst should possess both the abilities to dissociate water molecules and adsorb H on the surface active sites. On the other hand, HzOR under alkaline conditions is a four-electron transfer process [17–19]:

3 $$ {\text{N}}_{2} {\text{H}}_{4} + {\text{ OH}}^{ - } \to {\text{ N}}_{2} {\text{H}}_{3} * \, + {\text{ H}}_{2} {\text{O }} + {\text{ e}}^{ - } $$

4 $$ {\text{N}}_{2} {\text{H}}_{3} * \, + {\text{ OH}}^{ - } \to {\text{ N}}_{2} {\text{H}}_{2} * \, + {\text{ H}}_{2} {\text{O }} + {\text{ e}}^{ - } $$

5 $$ {\text{N}}_{2} {\text{H}}_{2} * \, + {\text{ OH}}^{ - } \to {\text{ N}}_{2} {\text{H}}* \, + {\text{ H}}_{2} {\text{O }} + {\text{ e}}^{ - } $$

6 $$ {\text{N}}_{2} {\text{H}}* \, + {\text{ OH}}^{ - } \to {\text{ N}}_{2} + {\text{ H}}_{2} {\text{O }} + {\text{ e}}^{ - } $$

Several intermediates and products are involved in the protonation processes of N_2_H_4_. Efficient HzOR electrocatalysts are required to have the ability to properly adsorb hydrazine molecules and promote the four consecutive deprotonation steps. Differently, an accelerated two-step HzOR mechanism was also proposed [20]. For example, Sun et al. concluded the HzOR mechanism on the Cu-doped Ni arrays [21]:

7 $${\text{NiCu}}* \, + {\text{ N}}_{2} {\text{H}}_{4} + \, 2{\text{OH}}^{ - } \to {\text{ NiCu }}{-}{\text{ N}}_{2} {\text{H}}_{2} * \, + {\text{ H}}_{2} {\text{O }} + \, 2{\text{e}}^{ - }$$

8 $$ {\text{NiCu }}{-}{\text{ N}}_{2} {\text{H}}_{2} * \, + \, 2{\text{ OH}}^{ - } \to {\text{ NiCu}}* \, + {\text{ N}}_{2} + \, 2{\text{H}}_{2} {\text{O }} + \, 2{\text{e}}^{ - } $$

A former slow two-electron transfer process to produce diazene intermediate and a latter fast two-electron transfer process to generate N_2_ are involved in this mechanism. However, a sufficient demonstration with corresponding characterization and electrochemical/calculation results of two-step HzOR mechanism has not been made so far, and the related parameters that can promote this two-step HzOR mechanism have not been discussed.

Recently, many Ni-based electrocatalysts are reported to exhibit excellent HER or HzOR electrocatalytic performance [22–26]. Among them, nickel selenide is promising to show bifunctional electrocatalytic activities on account of several electronic properties, including attraction of protons on the negatively charged Se atoms, favored hydrogen desorption by the weaker Se–H bonds and optimal adsorption N_2_H_y_ intermediates to promote the deprotonation processes in HzOR [27, 28]. Nevertheless, single-component nickel selenide generally exhibits undesirable electrocatalytic performance, let alone simultaneously accelerating complex reaction kinetics of both HER and HzOR. Heteroatom doping is one of the most effective strategies to improve the electrocatalytic performance of catalysts [29, 30]. The doping metal and non-metal heteroatoms can introduce atomic distortions, tune the lattice/electronic structure and create additionally exposed active sites for different electrocatalytic reactions. The adsorption energy for different reaction intermediates can be thus optimized to accelerate reaction kinetics. P-doping is reported to tune the electronic structure of nickel selenide and reduce the energy barrier for HER and anodic oxidation reaction [31]. Besides, metal atom-doping, especially Fe-doping, is reported to accelerate electron transfer in electrocatalytic reactions, thus accelerating reaction kinetics compared with the un-doped counterparts [32, 33]. Based on the above considerations, we developed ultrathin P/Fe co-doped NiSe_2_ nanosheets supported on modified Ni foam (P/Fe-NiSe_2_) synthesized through a two-step electrodeposition and subsequent selenization/phosphorous doping process. The as-synthesized electrocatalyst can thus catalyze favorable reaction kinetics for hydrazine-assisted water splitting. Low potentials of − 168 and 200 mV are required to reach 100 mA cm^−2^ for HER and HzOR, respectively. More importantly, based on electrochemical measurements, characterizations and density functional theory (DFT) calculations, a favorable “2 + 2” reaction mechanism with a two-step HER process and a two-step HzOR step on P/Fe-NiSe_2_ were fully proved, and the specific effect of P-doping on HzOR kinetics was investigated.

## Experimental Section

### Synthesis Procedures

Typically, a piece of commercial Ni foam (NF, 1 cm × 2 cm) was ultrasonically cleaned in hydrochloric acid, acetone, ethanol and deionized water to remove surface oxides and dried at 60 °C prior to the synthesis process. Then, a two-step galvanostatic electrodeposition was carried out in a two-electrode system using the pretreated NF as the working electrode and a Pt wire as the counter electrode. In the first step, 0.1 M NiCl_2_·6H_2_O and 2 M NH_4_Cl were added in to the electrolyte solution, and a cathode current density of 1 A cm^−2^ was applied for 10 min. The NF modified with Ni microspheres was obtained. In the second step, 0.75 M Ni(NO_3_)_2_·6H_2_O and 0.25 M FeSO_4_·7H_2_O were added in to the electrolyte solution and a cathodic current density of 10 mA cm^−2^ was applied for 200 s. The electrolyte solution was previously purged with N_2_ for 30 min to avoid the oxidation of iron salts. After each electrodeposition process, the prepared sample was rinsed with deionized water and dried at room temperature. The obtained Fe-doped NiOH supported on NF was named as Fe-NiOH. The Fe-NiOH was then heated at 300 °C for 90 min under N_2_ flow with 200 mg powder selenide and 100 mg NaH_2_PO_2_·H_2_O placed near the Fe-NiOH at the upstream side to obtain the final product P/Fe co-doped NiSe_2_ nanosheets supported on modified Ni foam (P/Fe-NiSe_2_). By adjusting the pyrolyzation temperature from 300 to 250 and 200 °C, P/Fe-NiSe_2_-250 and P/Fe-NiSe_2_-350 was obtained, respectively. For comparison, Fe-doped NiSe_2_ nanosheets supported on modified Ni foam (Fe-NiSe_2_) was prepared through a similar process to that of P/Fe-NiSe_2_ without the addition of NaH_2_PO_2_·H_2_O in the pyrolyzation process. NiSe_2_ nanosheets supported on modified Ni foam (NiSe_2_) was prepared through a similar process to that of Fe-NiSe_2_ without the addition of FeSO_4_·7H_2_O in the second electrodeposition step.

### Characterization

The Rigaku SmartLab diffractometer equipped with Cu-*K*α radiation was utilized to collect the X-ray diffraction (XRD) patterns. Scanning electron microscopy (SEM, Jeol JSM-7800F) and transmission electron microscopy (TEM, Jeol JEM-2800) were employed to characterize the structure and morphology of the synthesized materials. Utilizing a monochromatic Al-*K*α X-ray resource, X-ray photoelectron spectroscopy (XPS) was conducted via a Thermo Scientific ECSALAB 250Xi spectrometer. An electrochemical Nicolet iS50 spectrometer, complete with a VeeMAX III variable angle specular reflectance accessory as well as an electrochemical reaction cell, was utilized to characterize the electrochemical *in situ* Fourier transform infrared (FT-IR) spectroscopy.

### Electrochemical Measurements

A typical three-electrode system was employed to measure the prepared catalysts' electrochemical hydrogen evolution/hydrazine oxidation performance. Here, our prepared sample acted as the working electrode, a Hg/HgO electrode operated as the reference electrode, and a Pt wire was used as the counter electrode. On the other hand, the overall water/hydrazine splitting performance of the catalysts was assessed using a two-electrode setup where our prepared sample served as both the anode and cathode. In measuring hydrogen evolution/overall water splitting and hydrazine oxidation/overall hydrazine splitting, the electrolyte was 1.0 M KOH and 1.0 M KOH + 0.7 M N_2_H_4_, respectively.

## Results and Discussions

### Material Synthesis and Characterization

**Fig. 1:**
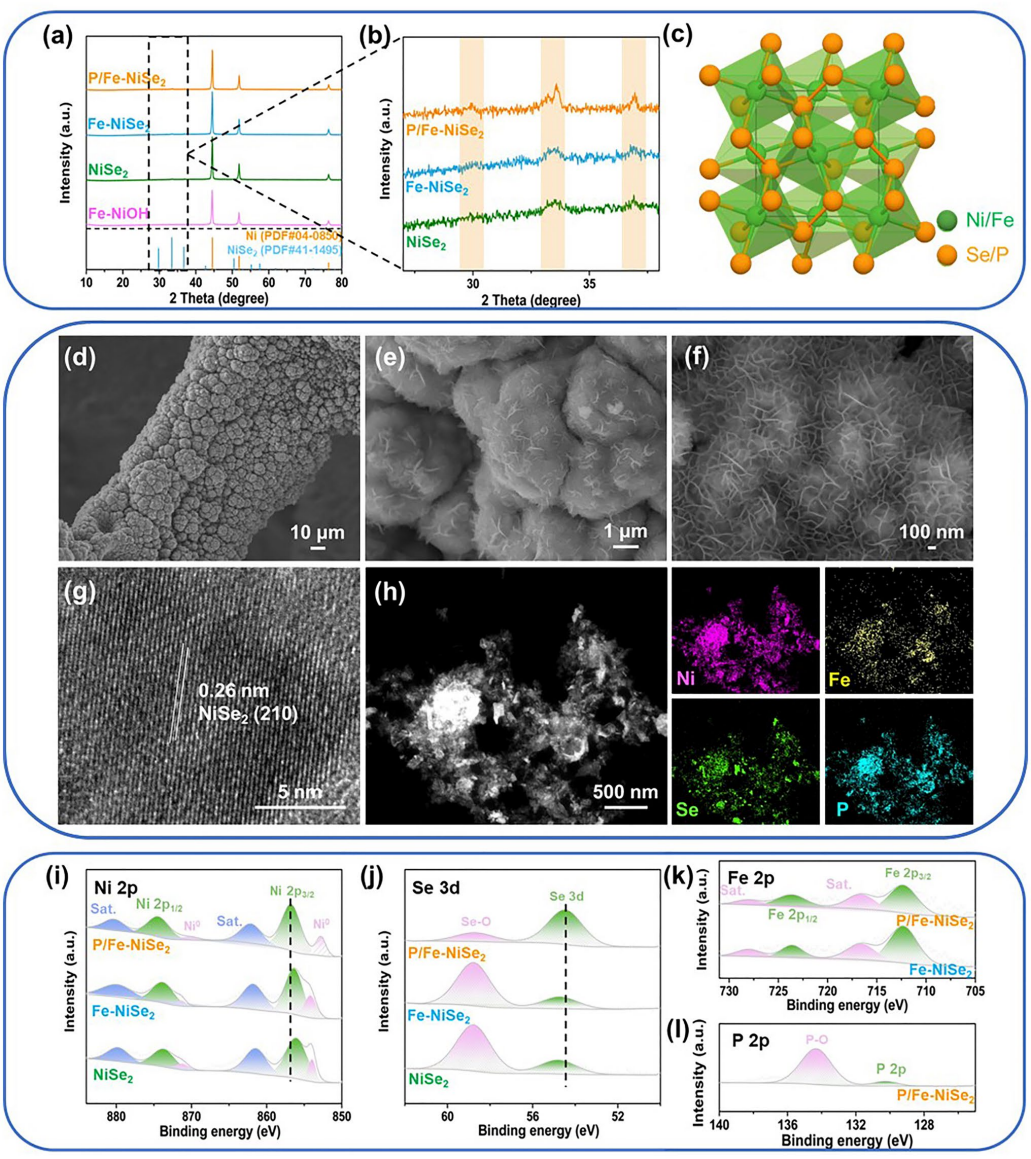
**a** XRD patterns of P/Fe-NiSe_2_, Fe-NiSe_2_, NiSe_2_ and Fe-NiOH. **b** Enlarged XRD patterns of P/Fe-NiSe_2_, Fe-NiSe_2_, NiSe_2_ from 27 to 38 degree. **c** Crystal structure of P/Fe-NiSe_2_. **d-f** SEM images of P/Fe-NiSe_2_. **g** High-resolution TEM image of P/Fe-NiSe_2_. **h** STEM image and the corresponding Ni, Fe, Se, P elemental mappings of P/Fe-NiSe_2_. XPS characterization. **i** Ni 2*p* and **j** Se 3*d* of P/Fe-NiSe_2_, Fe-NiSe_2_ and NiSe_2_. **k** Fe 2*p* of P/Fe-NiSe_2_ and Fe-NiSe_2_. **l** P 2*p* of P/Fe-NiSe_2_

The ultrathin P/Fe co-doped NiSe_2_ nanosheets supported on modified Ni foam were synthesized through a two-step electrodeposition process and subsequent pyrolysis process for selenization and phosphorous doping, as shown in Scheme S1. In order to increase the roughness of NF and expose more active sites, the commercial NF with smooth surface (Fig. S1) was modified with Ni microspheres (Fig. S2) via a bubble template method in the first electrodeposition step (Fig. S3). Subsequently, ultrathin Fe-doped NiOH nanosheets were vertically grown on the surface of Ni microspheres during the second electrodeposition process (Fig. S4) to prepare the Fe-NiOH (Fig. S5). The as-prepared Fe-NiOH was then subjected to a low-temperature pyrolysis to simultaneously achieve selenization and phosphorous doping to obtain P/Fe-NiSe_2_. For comparison, Fe-NiSe_2_ and NiSe_2_ were also prepared. The X-ray diffraction (XRD) patterns of P/Fe-NiSe_2_ and Fe-NiSe_2_ (Fig. 1a, b) show diffraction peaks indexed to NiSe_2_ (PDF#41-1495), indicating that the original NiOH was transformed into NiSe_2_ with P and Fe dopants (Fig. 1c). Scanning electron microscopy (SEM) images of P/Fe-NiSe_2_ (Fig. 1d, f) show well retention of original morphology of Fe-NiOH after the selenization and P-doping during the pyrolysis process. Fe-NiSe_2_ and NiSe_2_ show similar morphology (Figs. S6 and S7), indicating that only composition differences exist between them. The high-resolution TEM image (Fig. 1g) shows the lattice fringes with interplanar spacing of 0.26 nm in the observational region, corresponding to NiSe_2_ (2 1 0). Furthermore, the homogeneous distribution of doping P and Fe into the NiSe_2_ lattice is demonstrated by scanning transmission electron microscopy (STEM) image and the corresponding elemental mappings of Ni, Fe, Se and P, proving the formation of P/Fe-co-doped NiSe_2_ (Fig. 1h).

The elemental compositions and the effect of P/Fe-doping on the surface chemical states of P/Fe-NiSe_2_ were investigated by X-ray photoelectron spectroscopy (Fig. 1i, j, k, l). The Ni 2*p* spectrum (Fig. 1i) of P/Fe-NiSe_2_ shows two core-level peaks at 874.4 and 856.7 eV with their corresponding satellite peaks at 880.3 and 862.1 eV, which are ascribed to Ni^2+^ 2*p*_1/2_ and Ni^2+^ 2*p*_3/2_, respectively [34]. Two peaks at 870.3 and 852.8 eV are assigned to the metallic state of Ni. Note that the binding energies of Ni 2*p*_2/3_ and the corresponding satellite peak of P/Fe-NiSe_2_ exhibit an obviously positive shift of approximately 0.4 and 0.6 eV compared with Fe-NiSe_2_ and NiSe_2_, respectively. That indicates the positively charged metal atoms with decreased localization electron density [35]. By contrast, the Se 3*d* peak of P/Fe-NiSe_2_ (Fig. 1j) at 54.4 eV shows negative shift compared with Fe-NiSe_2_ and NiSe_2_, indicating that Se atoms are partially negatively charged after the incorporation of P and Fe atoms. These results demonstrate an electron distribution on Ni–Se bonds after heteroatom doping, contributing to decreased electron densities around Ni atoms and increased electron densities around Se atoms, which might optimize the adsorption of proton and hydrazine [36]. The other main peak in Se 3*d* spectrum at 58.7 eV is ascribed to Se–O bonding, which may be derived from surface oxidation of NiSe_2_. The Fe 2*p* spectrums (Fig. 1k) of P/Fe-NiSe_2_ and Fe-NiSe_2_ show similar characteristic peaks of 2*p*_1/2_ (~ 723.6 eV) and 2*p*_3/2_ (~ 712.3 eV) and their corresponding satellite peaks at approximately 728.0 and 716.6 eV, respectively [37]. The P 2*p* spectrum (Fig. 1l) of P/Fe-NiSe_2_ exhibits two main peaks at 130.3 and 134.3 eV, which are assigned to P 2*p* and P–O bonding, respectively [38]. Based on the aforementioned discussions, P and Fe atoms are successfully incorporated into the cubic NiSe_2_ lattice to form P/Fe-co-doped ultrathin NiSe_2_ nanosheets supported on modified Ni foam as a potential electrocatalyst for overall hydrazine splitting.

### Electrochemical Measurements

Electrocatalytic HER and HzOR performances of the synthesized electrocatalysts were tested in a three-electrode system. First, linear sweep voltammetry (LSV) curves of P/Fe-NiSe_2_ for HzOR in 1.0 M KOH with different hydrazine concentrations were compared to identify the optimum hydrazine concentration (Fig. S8). The reactive current densities obviously increase with N_2_H_4_ up to 0.7 M. Thus, the electrocatalytic HzOR performance of the synthesized electrocatalysts was all tested in 1.0 M KOH with addition of 0.7 M N_2_H_4_. In order to optimize the electrocatalytic performances for overall hydrazine splitting, the samples of P/Fe-NiSe_2_-250 and P/Fe-NiSe_2_-350 varying pyrolysis temperature to 250 and 350 °C were also synthesized and evaluated. Figure S9 shows that P/Fe-NiSe_2_ employing 300 °C as the pyrolysis temperature exhibits the best bifunctional HER/HzOR performance. Therefore, P/Fe-NiSe_2_ with the optimal synthesis condition was used in all the subsequent electrochemical measurements.

**Fig. 2:**
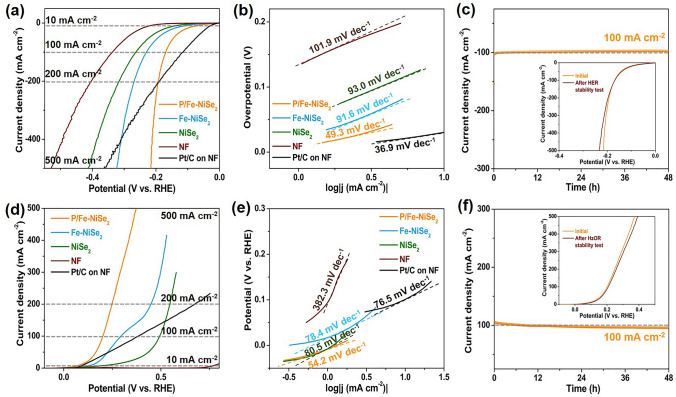
Electrocatalytic hydrogen evolution/hydrazine oxidation performances of P/Fe-NiSe_2_ and other comparison samples Fe-NiSe_2_, NiSe_2_, NF and Pt/C on NF. **a** Polarization curves for HER and corresponding **b** Tafel plots. **c** Amperometry for HER stability of P/Fe-NiSe_2_. Inset: LSV measurement before and after HER stability test. **d** Polarization curves for HzOR and corresponding **e** Tafel plots. **f** Amperometry for HzOR stability of P/Fe-NiSe_2_. Inset: LSV measurement before and after HzOR stability test

**Fig. 3:**
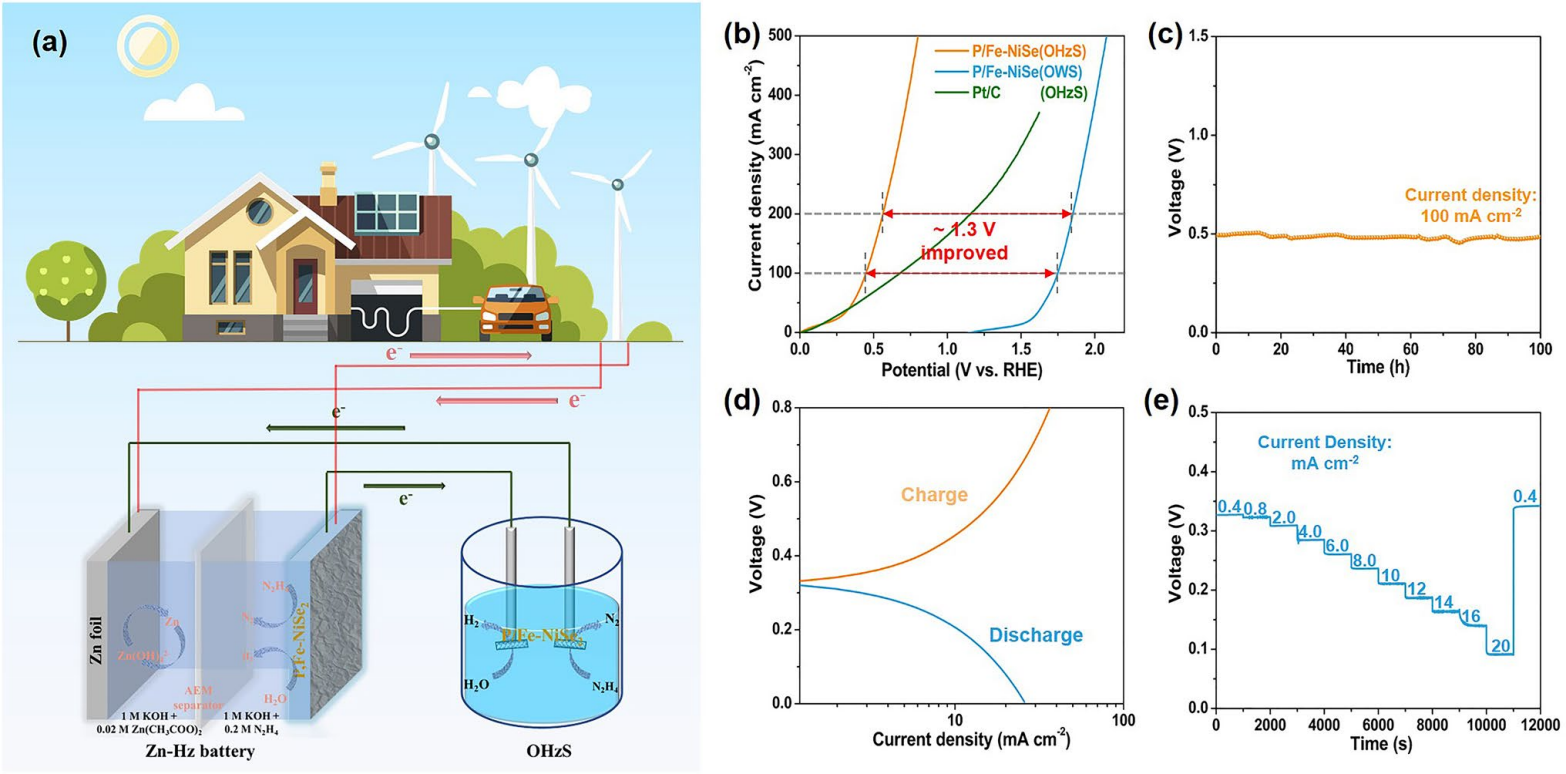
**a** Schematic illustration of the combination of OHzS, Zn-Hz battery and renewable energy. **b** Polarization curves for hydrazine-assisted water electrolysis. **c** Long-term stability test at a fixed current density of 100 mA cm^−2^. **d** Polarization curves and **e** galvanostatic discharge/charge curves at different current densities for Zn-Hz battery

Figure 3a shows the LSV curves of P/Fe-NiSe_2_ and other comparison samples Fe-NiSe_2_, NiSe_2_, bare NF and commercial Pt/C on NF for HER. P/Fe-NiSe_2_ exhibits obviously enhanced electrocatalytic HER activity with decreased onset overpotential and increased current densities. The overpotential required for current density of 10 mA cm^−2^ is only 74 mV, which is superior to those of Fe-NiSe_2_ (110 mV), NiSe_2_ (141 mV), bare NF (230 mV) and slightly higher than that of commercial Pt/C (31 mV). This value of 74 mV is also lower than those of many recently reported efficient electrocatalysts (Fig. S10 and Table S1). The corresponding Tafel slope of P/Fe-NiSe_2_, Fe-NiSe_2_, and NiSe_2_ is 49.3, 78.4 and 80.5 Mv dec^−1^, respectively, indicating faster reaction kinetics on P/Fe-NiSe_2_ for rapid hydrogen evolution (Fig. 3b). By measuring cyclic voltammograms (CVs) at different scan rates (Fig. S11), the double-layer capacitance (*C*_dl_) of P/Fe-NiSe_2_ is calculated to be 77.7 mF cm^−2^, which is close to those of Fe-NiSe_2_ (66.1 mF cm^−2^) and NiSe_2_ (59.3 mF cm^−2^) and much higher than that of pristine Ni foam (1.9 mF cm^−2^), suggesting that more active sites can be exposed. The polarization curves using ECSA-normalized current densities (Fig. S13) reveal the intrinsic optimization of electrocatalytic active sites on P/Fe-NiSe_2_ with P and Fe doping. Besides, the H_2_ turn over frequency (TOF) per surface site was also calculated to further investigate the intrinsic HER activities of these electrocatalysts. The calculated TOFs are plotted against potential in Fig. S14. The TOF values for P/Fe-NiSe_2_ are averagely more than 5 times larger than those for Fe-NiSe_2_ and NiSe_2_. Specifically, the experimental TOFs of P/Fe-NiSe_2_, Fe-NiSe_2_ and NiSe_2_ are 4.5, 2.4 and 1.3 s^−1^ at − 0.1 V vs. RHE, respectively. These observations suggest that P/Fe-NiSe_2_ possesses enhanced HER activity, which is mainly attributed to intrinsically optimized active sites with favorable reaction kinetics, rather than different active surface areas. The long-term stability of P/Fe-NiSe_2_ was also evaluated by amperometry curves at a constant applied potential (Fig. 2c). The P/Fe-NiSe_2_ can reserve the original current density of 100 mA cm^−2^ for over 24 h with less than 5% loss of current density. The LSV plots measured after stability test further demonstrate nearly unchanged electrocatalytic HER activity (inset Fig. 2c). In addition, the post-HER P/Fe-NiSe_2_ were also investigated by SEM (Fig. S15), XRD (Fig. S16) and XPS analysis (Fig. S17), showing well retention of structure and composition with negligible change.

In addition to the excellent electrocatalytic HER performance, P/Fe-NiSe_2_ also exhibits outstanding electrochemical HzOR performance in N_2_H_4_-containing 1.0 M KOH. As shown in the polarization curves in Fig. 2d, the P/Fe-NiSe_2_ shows the best electrocatalytic HzOR performance with advantageous overpotentials and enhanced reactive current densities. Specifically, the P/Fe-NiSe_2_ can accomplish a current density of 100 mA cm^−2^ at a low potential of 200 mV, which makes it superior to Fe-NiSe_2_ (294 mV), NiSe_2_ (493 mV), commercial Pt/C catalyst and other recently reported efficient HzOR electrocatalysts (Fig. S10 and Table S2). As shown in Fig. 2e, P/ Fe-NiSe_2_ shows the smallest Tafel slope of 54.2 mV dec^−1^, indicating an effective kinetical improvement after heteroatom doping, compared with Fe-NiSe_2_ (78.4 Mv dec^−1^) and NiSe_2_ (80.5 mV dec^−1^). The ECSA-normalized polarization curves of different samples for HzOR are shown in Fig. S18. It can be observed that P/Fe-NiSe_2_ shows lower overpotentials to obtain different current densities than Fe-NiSe_2_ and NiSe_2_, revealing intrinsically enhanced HzOR activity of P/Fe-NiSe_2_. The electrochemical stability for HzOR is a crucial index to assess the performance of electrocatalysts. The long-term stability of P/Fe-NiSe_2_ was evaluated by an amperometry test at a fixed potential. As shown in Fig. 2f, P/Fe-NiSe_2_ can sustain a high current density of 100 mA cm^−2^ for over 24 h with minimal loss of current density and shows a good LSV overlapping with no obvious performance decay before and after the long-term stability test (inset Fig. 2f). Furthermore, the SEM (Fig. S19) and XRD (Fig. S20) analysis of the post-HzOR sample demonstrates respectable preservation of original morphology and crystallinity. In another concern, XPS analysis of P/Fe-NiSe_2_ shows some modification after long-term HzOR stability test (Fig. S21). Specifically, the high-resolution Ni 2*p* XPS spectrum of post-HzOR P/Fe-NiSe_2_ sample (Fig. S21a) shows the disappearance of characteristic peaks assigned to metallic Ni and Se 3*d* spectrum (Fig. S21b) shows an intensity increase at 58.7 eV (assigned to Se-O), which can be attributed to the partial oxidation of NiSe_2_ during HzOR process. No other changes of structure can be detected on the post-HzOR P/Fe-NiSe_2_, indicating the satisfactory stability of the electrode.

### Overall Hydrazine Splitting and Zn-Hz Battery Performance

Considering the excellent electrocatalytic HER and HzOR performance of P/Fe-NiSe_2_, is promising in overall hydrazine splitting (OHzS) and other efficient devices (Fig. 3a). As shown in Fig. 3b, ultralow potentials are required for high current densities for hydrazine-assisted water electrolysis with decreased applied voltages of about 1.3 V compared with traditional water electrolysis, which could be powered by a commercial solar panel without additional applied potential under sunlight (Fig. S22). This electrolysis performance is better than that of many other recently reported electrocatalysts (Table S3). In addition, the OHzS device shows remarkably stable performance at a high current density of 100 mA cm^−2^ for at least 100 h with negligible fluctuation of applied voltages (Fig. 3c). The faradaic efficiency was demonstrated to be approximately 100%, proving a high selectivity of P/Fe-NiSe_2_ in hydrazine-assisted water electrolysis (Fig. S23). Zn-hydrazine (Zn-Hz) battery is a recently reported hydrazine-assisted energy conversion device, which is assembled with Zn foil as anode and bifunctional HER/HzOR electrocatalyst as cathode, separated by anion-exchange membrane (AEM) as separator [39]. At the anode, electrons transfer with the cycle of metal Zn and Zn(OH)_4_^2−^. At the cathode, hydrogen is produced by the electrocatalytic HER during the discharge process, while nitrogen is produced through the electro-oxidation of hydrazine during the charge process. As shown in Fig. S24, P/Fe-NiSe_2_-assembled Zn-Hz battery could maintain an open circuit voltage of 0.330 V for more than 1 h, proving the feasibility in Zn-Hz battery. Figure 3d shows the discharge and charge polarization curves with small voltage gaps, further verifying the good feasibility. Figure 3e shows that the Zn-Hz battery could discharge stably at various current densities, especially at a relatively high current density of 20 mA cm^−2^. The Zn-Hz battery could charge after long-term discharging, suggesting its good rechargeability. Based on the discharge voltage of 327 mV and the charge voltage of 337 mV at 0.4 mA cm^−2^, the energy efficiency of this assembled Zn-Hz battery can be calculated to be more than 97%. Besides, the assembled Zn-Hz battery could operate stably for more than 300 cycles (100 h) at 5 mA cm^−2^ without obvious voltage gap change, proving its excellent stability in the applied environment (Fig. S25). Thus, the P/Fe-NiSe_2_-assembled Zn-Hz battery is promising to be a transfer station between intermittent renewable energy and P/Fe-NiSe_2_-assembled water electrolyzer (Fig. 3a). The intermittent renewable energy like wind and solar energy can be stored in Zn–Hz battery to sustain the stable operation of water electrolysis device, which has great value in practical application.

### Insight into the Accelerated Kinetics

Given the desirable bifunctional electrocatalytic performance, we applied further electrochemical analysis, characterization and theoretical calculation to investigate the favorable reaction kinetics of P/Fe-NiSe_2_. For HER, the electrochemical impedance spectroscopy (EIS) measurements (Fig. S26) show that the samples with Fe-doping feature drastic fast charge transfer relative to NiSe_2_ with low charge transfer resistance (*R*_ct_) (Fig. S27). The electron transfer and electrocatalytic kinetics during HER process were further explored by *Operando* EIS at different potentials (Fig. S28). As shown in Fig. S28a-c, the Nyquist spectra at the low potentials show infinite *R*_ct_ with nearly vertical lines. Evident semicircles arise at higher applied potentials, suggesting that the electrocatalytic HER has appeared. P/Fe-NiSe_2_ exhibits lower applied potential for the appearance of evident semicircle than Fe-NiSe_2_ and NiSe_2_, which is in line with the lower overpotentials in polarization curves, indicating higher electrocatalytic HER activity of P/Fe-NiSe_2_. In addition, the change of phase angle (degree) with frequency is shown in the Bode phase plots (Fig. S28d-f). It is reported that the peaks of phase angle at low-frequency area can be attributed to the reaction charge transfer at the interface, which can be inspection parameter of hydrogen evolution reaction kinetics [40, 41]. It can be observed that P/Fe-NiSe_2_ and Fe-NiSe_2_ show similar decrease tendency of phase angle, which is much quicker than that of NiSe_2_ without Fe-doping, suggesting faster reaction charge transfer at the interface and accelerated HER kinetics because of Fe doping. Besides, the temperature-dependent kinetic analysis (Fig. S29) shows a lower apparent activation energy value of for P/Fe-NiSe_2_ (38.9 kJ mol^−1^) compared to those of Fe-NiSe_2_ (68.3 kJ mol^−1^) and NiSe_2_ (74.9 kJ mol^−1^), proving the optimization in enthalpy of hydrogen evolution process due to the introduction of P atoms, reducing the kinetic barrier for the HER intermediates and accelerating the hydrogen evolution process [42, 43]. In situ Fourier transform infrared (FTIR) spectroscopy was employed to further investigate the specific effect of P doping at the molecular level. As shown in the absorbance spectra collected at − 0.1 V vs. RHE (Fig. S30), the most prominent peak at around 1640 cm^−1^ (green area in Fig. S30) is assigned to the OH bending mode, which represents the change of OH^−^ concentration on the reaction interface related to the hydrogen evolution process [44–46]. The negative-going band for P/Fe-NiSe_2_ is obviously more intense than those for Fe-NiSe_2_ and NiSe_2_, demonstrating the accelerated HER kinetics by incorporation of P element.

**Fig. 4:**
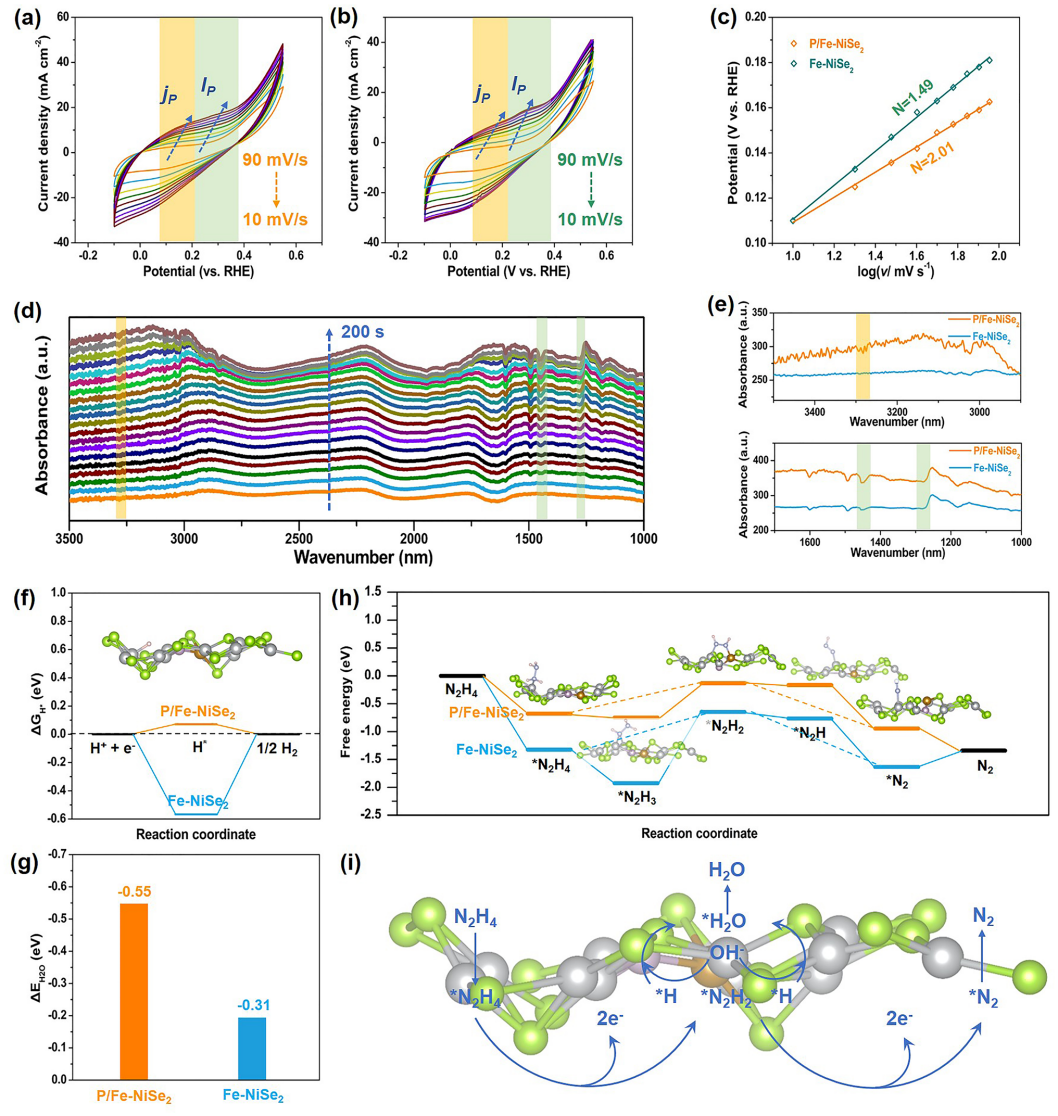
CVs obtained with 10 mM hydrazine in 1.0 M KOH at different scan rates over **a** P/Fe-NiSe_2_ and **b** Fe-NiSe_2_. **c** HzOR peak potential plots versus log(*v*). **d, e** Electrochemical in situ FTIR spectroscopy over P/Fe-NiSe_2_ and Fe-NiSe_2_ for HzOR. **f** Gibbs free energy diagram for H adsorption. **g** H_2_O adsorption energy. **h** Gibbs energy profiles for HzOR intermediates and the most stable configurations of each adsorbed intermediate for P/Fe-NiSe_2_. **i** Schematic illustration of the proposed HzOR processes over P/Fe-NiSe_2_

For HzOR, to prove the two-step reaction mechanism hypothesis and investigate the specific effect of P doping on the HzOR reaction kinetics, CV curves of P/Fe-NiSe_2_ and Fe-NiSe_2_ were measured in 10 mM N_2_H_4_-containing 1.0 M KOH varying scan rates from 10 to 90 mV s^−1^ (Fig. 4a, b). It can be observed that the nickel hydroxide-relevant anodic peak and hydrazine oxidation peak appear. The nickel hydroxide-relevant anodic peak current densities (*I*_p_) and hydrazine oxidation peak current densities (*j*_p_) increase and show a well-linear relationship with the increasing square root of scan rate (Fig. 4c), which suggest a diffusion-controlled HzOR process at low N_2_H_4_ concentrations. Specifically, *j*_p_ and its peak potential (*E*_p_) have a quantitative relationship with the square root of scan rate, which can be described by the following equations [21, 47]:

9 $$ j_{p} = \, 3.01 \times 10^{5} n[(1 \, - \alpha )n_{\alpha } ]^{1/2} {\text{AD}}^{1/2} {\text{Cv}}^{1/2} $$

10 $$ E_{p} = \, [2.303{\text{RT}}/(1 \, {-}\alpha )n_{\alpha } F]\;{\text{log}}v + K $$

Here, *n* is the total electron transfer number, *α* is the electron transfer coefficient, *n*_*α*_ is the electron transfer number in the rate-determining step, *A* is the electrode geometric area, *D* is the diffusion coefficient, *C* is the bulk hydrazine concentration and *v* is the scan rate. *R*, *T*, *F* and *K* are the gas constant (8.314 J K^−1^ mol^−1^), temperature, Faraday constant (96,485 C mol^−1^) and the constant, respectively. In this way, *n*_*α*_ of P/Fe-NiSe_2_ and Fe-NiSe_2_ are calculated to be 2.01 (approximately equal to 2) and 1.49, respectively. Thus, the rate-determining step of HzOR process on P/Fe-NiSe_2_ involves a two-electron transfer process to produce a diazene intermediate, while that on Fe-NiSe_2_ involves a mixture of one-electron transfer process and two-electron transfer process. This can well explain the accelerated HzOR reaction kinetics on P/Fe-NiSe_2_, which may be attributed to the positively charged Ni sites with reduced energy barrier for the dehydrogenation process in HzOR [3]. In situ Fourier transform infrared (FTIR) spectroscopy was employed to further investigate the reaction kinetics on modified active sites with the incorporation of P at the molecular level. When employing in situ FTIR spectroscopy over P/Fe-NiSe_2_ in N_2_H_4_-containing KOH solution at 0.2 V vs. RHE for 200 s, several peaks assigned to N_2_H_*y*_ (1 ≤ *y* ≤ 4) intermediates for HzOR increase and finally reach a stable state, including N–H stretching at around 3280 cm^−1^, H–N–H bending at around 1450 cm^−1^ and -NH_2_ wagging at around 1280 cm^−1^ (Fig. 4d) [48–50]. As shown in Fig. 4e, the amounts of different HzOR reaction intermediates of P/Fe-NiSe_2_ are higher than that of Fe-NiSe_2_, further revealing the accelerated reaction kinetics over P/Fe-NiSe_2_ with modified electronic structure of Ni sites by the introduction of P element. Notably, compared with other peaks, the -NH_2_ wagging peaks of P/Fe-NiSe_2_ and Fe-NiSe_2_ show a smaller absorbance gap, which can be attributed to different reaction mechanisms, specifically, the lack of N_2_H_3_ intermediate in the two-step hydrazine oxidation process for P/Fe-NiSe_2_.

To investigate the origins of the superior bifunctional activity of P/Fe-NiSe_2_ and the specific effect of P doping on the HzOR reaction kinetics, density functional theory (DFT) calculations were further performed (Fig. S31). Δ ${G}_{{\mathrm{H}}^{*}}$ is generally considered as the key parameter to evaluate the electrocatalytic hydrogen evolution performance, and a near thermoneutral Δ ${G}_{{\mathrm{H}}^{*}}$ is favorable for the adsorption and desorption of hydrogen [3]. The Gibbs free energy of the adsorbed H* (Δ ${G}_{{\mathrm{H}}^{*}}$) on P/Fe-NiSe_2_ and Fe-NiSe_2_ is 0.07 and − 0.57 eV, respectively (Fig. 4f), indicating an optimized H bonding on P/Fe-NiSe_2_. Furthermore, water adsorption on the electrocatalytic surface is also critical for HER. A lower water adsorption energy ($\Delta E_{{{\text{H}}_{2} {\text{O}}}}$) on P/Fe-NiSe_2_ was calculated to be − 0.55 eV, compared with that of − 0.32 eV on Fe-NiSe_2_ (Fig. 4g), proving an accelerated water adsorption process on P/Fe-NiSe_2_. For HzOR, the electrocatalytic performance depends on the free energies of each reaction intermediates in consecutive dehydrogenation steps. Figure 4h and Table S4 show the calculated Gibbs free energies for HzOR intermediates on P/Fe-NiSe_2_. The solid line and the dotted line represent the traditional four-step and the suggested two-step HzOR process, respectively. In terms of the four-step dehydrogenation process, the RDS for both P/Fe-NiSe_2_ and Fe-NiSe_2_ is the dehydrogenation from *N_2_H_3_ to *N_2_H_2_, in which the free energy difference value for P/Fe-NiSe_2_ (0.62 eV) is lower than that for Fe-NiSe_2_ (1.28 eV), proving an accelerated hydrazine oxidation process on P/Fe-NiSe_2_. Specifically, the free energy difference value between the first and the second dehydrogenation process is relatively high (1.89 eV), indicating that the electro-oxidation of hydrazine on Fe-NiSe_2_ involves a former fast electron transfer process to produce *N_2_H_3_ and a latter low electron transfer process to produce *N_2_H_2_, which is consistent with the FTIR results. Additionally, the free energy difference between the first dehydrogenation process and the two-electron process to produce *N_2_H_2_ is also high (1.28 eV), proving that the two-electron transfer process is relatively unfavored on Fe-NiSe_2_. On the contrast, after P doping, these two free energy difference values dramatically decrease to be 0.69 and 0.62 eV, indicating a favored two-electron transferred process from *N_2_H_4_ to *N_2_H_2_ over P/Fe-NiSe_2_. Similarly, the two-electron transferred process from *N_2_H_2_ to *N_2_ is also preferable on P/Fe-NiSe_2_, composing a two-step HzOR process, which can be illustrated by Fig. 4i. Thus, the bifunctional electrocatalytic HER and HzOR activity and a synthetically favorable “2 + 2” reaction mechanism with a two-step HER process and a two-step HzOR process of P/Fe-NiSe_2_ have been proved, making it promising to be used in overall hydrazine splitting.

## Conclusions

In summary, we have developed ultrathin P/Fe co-doped NiSe_2_ nanosheets supported on modified Ni foam as highly efficient bifunctional HER and HzOR electrocatalysts for hydrazine-assisted water splitting. The prepared P/Fe-NiSe_2_ can achieve a high current density of 100 mA cm^−2^ at potentials of − 168 and 200 mV for HER and HzOR, respectively. The specific effects of P and Fe doping have been investigated in view of accelerated charge transfer and optimized reaction kinetics based on electrochemical measurements, characterizations and DFT calculations. A favorable “2 + 2” reaction mechanism is thus concluded with a two-step HER process and a two-step HzOR step. This work not only reports an efficient bifunctional electrocatalyst for overall hydrazine splitting, but also provides a deeper mechanistic understanding of the heteroatom doping for HER/HzOR and may stimulate creativity for the development of advanced electrocatalysts for hydrogen production.

### Supplementary Information

Below is the link to the electronic supplementary material.

Supplementary file1 (PDF 1917 kb)
