# Supplementary material for: Heteroatom-Induced Accelerated Kinetics on Nickel Selenide for Highly Efficient Hydrazine-Assisted Water Splitting and Zn-Hydrazine Battery
Source: Nanomicro Lett. 2023 Jun 19;15:155. doi: 10.1007/s40820-023-01128-z (PMC10279626; doi:10.1007/s40820-023-01128-z)
Supplement: Supplementary file 1 — Supplementary file1 (PDF 1917 kb) [file 40820_2023_1128_MOESM1_ESM.pdf]

Supporting Information for

Heteroatom-Induced Accelerated Kinetics on Nickel Selenide for Highly Efficient Hydrazine-Assisted Water Splitting and Zn-Hydrazine Battery

Hao-Yu Wang [1], Lei Wang [1], Jin-Tao Ren [1], Wen-Wen Tian [1], Ming-Lei Sun [1] and Zhong-Yong Yuan [1, 2, *]

[1] School of Materials Science and Engineering, Smart Sensing Interdisciplinary Science Center, Nankai University, Tianjin 300350, P. R. China

[2] Key Laboratory of Advanced Energy Materials Chemistry (Ministry of Education), Nankai University, Tianjin 300071, P. R. China

*Corresponding author. E-mail: zyyuan@nankai.edu.cn (Zhong-Yong Yuan)

S1 Experimental Section

S1.1 Materials

Nickel foam (99%) used in the study was obtained from Shanxi Lizhiyuan Technology Co., Ltd. Hydrochloric acid (HCl, 36.0%～38.0%) was obtained from Yongfei Chemical Reagent Co., Ltd. Sodium hypophosphite ($NaH_2PO_2·H_2O$, 99.0%) and acetone (99.5%) were received from Tianjin Chemical Reagent Supply and Marketing Co., Ltd. Ethyl Alcohol (99.7%) was purchased from Concord Technology Co., Ltd. Deionized water was purchased from Tianjin Huaxun Medical Technology Co., Ltd. Ammonium chloride (NH_4Cl, 99.5%), sodium chloride (NaCl, 99.5%) and potassium hydroxide (KOH, 85%) were purchased from Tianjin Bohua Chemical Reagent Co., Ltd. Nickel chloride hexahydrate ($NiCl_2·6H_2O$, 98.0%) was received from Tianjin Chemical Reagent wholesale Co., Ltd. Powder selenium (Se 99.5%) was purchased from Beijing Chaoyang Zhonglian Chemical Reagent Factory. 20 wt% Pt/C powders were purchased from Shanghai Hesen Electric Co., Ltd. Nickel nitrate ($Ni(NO_3)_2·6H_2O$, 98%) and hydrazine hydrate ($N_2H_4·H_2O$, 80%) was purchased from Tianjin Damao Chemical Reagent Co., Ltd.

S1.2 Electrochemical Measurements

For the preparation of Pt/C on NF control electrode, 5 mg of commercial Pt/C electrocatalyst (20 wt %) was dispersed into 480 μL deionized water/isopropanol (1:4) and 20 μL of Nafion solution (5 wt%) with sonication to obtain homogeneous catalyst ink. Then, 50 μL of catalyst ink was transferred onto one piece of Ni foam with the Pt loading mass of 200 μg cm^{-2}.

To fairly compare the performance of various catalysts, all the polarization curves

were corrected for ohmic losses throughout the system, which include the wiring, substrate, catalyst material, and solution resistances. All these resistances constitute the series resistance (R_s) of the measurement. The R_s can be obtained from an EIS Nyquist plot as the first intercept of the main arc (corresponding to the electrode-electrolyte interface) with the real axis. For all measurements, the values of R_s are low and consistent. Then the iR-corrected data is given by $E_{\mathrm{Corrected}} = E_{\mathrm{Raw}} - iR_s$.

The Tafel slope was calculated according to the Tafel equation of $\eta = b \cdot \log(J/J_0)$, where η, b, J and J_0 indicate the overpotential, Tafel slope, current density, and exchange current density, respectively.

The electrochemical double layer capacitance (C_{dl}) was determined by the cyclic voltammograms under the scan rate from 10 to 30 mV s^{-1} in a narrow non-Faradaic potential range. The double layer capacitance C_{dl} is determined from CV measurements based on the equation $C_{\mathrm{dl}} = (j_a - j_c)/(2*v) = (j_a + |j_c|)/(2*v) = \Delta j/(2*v)$, in which j_a and j_c are the anodic and cathodic current density, respectively, recorded at the middle of the selected potential range, and v is the scan rate. The C_{dl} can be further converted into electrochemical active surface area (ECSA) using roughness factor (r_f):

$$r_f = C_{\mathrm{dl}}\ (\mathrm{mF\ cm^{-2}})/C_{\mathrm{dl,ideal}}\ (\mathrm{mF\ cm^{-2}})$$

$$j_{\mathrm{ECSA}} = j/r_f$$

where $C_{\mathrm{dl,ideal}}$ is the double layer capacitance of an ideally flat electrode (specific C_{dl}), which is usually taken as $C_{\mathrm{dl,ideal}} = 0.04$ mF cm^{-2} for alkaline media. j_{ECSA} is the current density normalized by ECSA.

The electrochemical impedance spectroscopy (EIS) tests were performed at different potentials in the frequency range from 0.01 to 100000 Hz with the amplitude of 5 mV.

The TOF values are calculated via the following equation:

$$TOF = \frac{|j|A}{mFN}$$

where $|j|$ is the current density at a fixed voltage during the LSV measurement in 1.0 M solution, A stands for the area of the electrode (0.5 cm^{-2}) and F is the Faradic constant (96485 C mol^{-1}). N represents the quantity of active sites, which is calculated by measuring CV curves at 0.05 V s^{-1}. A factor of 1/m is introduced, taking into account that m electrons are consumed to form one H_2 molecule from water.

The pre-exponential factor (A_{app}) and apparent activation energy (E_{app}) at fixed overpotentials can be calculated based on Arrhenius equation [S1]:

$$i_{\mathrm{ECSA}} = A_{\mathrm{app}} \exp(E_{\mathrm{app}}/RT)$$

Herein, i_{ECSA} presents the ECSA normalized current density, R is the ideal gas constant (8.314 J K^{-1} mol^{-1}) and T stands for the Kelvin temperature (K). Therefore, E_{app} values at different applied potentials can be obtained by using the following equation [S2]:

$$|\partial(\log_{10} i_{ECSA})/\partial(1/T)| = -E_{app}/2.303R$$

Faradaic efficiency was calculated by comparing the volume of experimentally quantified gas (water-gas displacing method at a current density of 200 mA cm^{-2}) with theoretically calculated gas, $\eta_{Faradaic\ efficiency} = (V_{experimental}/V_{theoretical}) \times 100\%$; the theoretical volume of evolving gases can be calculated using the equation: $V_{theoretical} = (I \times t \times V_m)/(n \times F)$, where I is the current measured in the experiment, t is the recorded time, V_m is the molar volume of H_2 or O_2 in 1/mol, n is the number of electrons needed for 1 mol H_2 or O_2, and F is the Faraday's constant (96,485 C/mol).

1.3 Density Functional Theory Calculations

Density functional theory (DFT) based first-principles calculations are performed using the projected augmented wave (PAW) [S3] method implemented in the Vienna ab initio simulation package (VASP) [S4, S5]. The Kohn-Sham one-electron states are expanded using the plane-wave basis set with a kinetic energy cutoff of 500 eV. The Perdew-Burke-Ernzerhof (PBE) exchange-correlation functional within the generalized gradient approximation (GGA) is employed [S6]. To study the mechanistic details of surface reactions, the $NiSe_2$ (210) surface is modeled by a periodic slab repeated in 1×2 surface unit cell. P-doping is simulated by substituting Se with P. Fe-doping is simulated by substituting Ni with Fe. The Brillouin-zone (BZ) integration is carried out using the Monkhorst-Pack sampling method with a density of 2×2×1 for the geometry optimizations [S7]. A sufficiently large vacuum region of 15 Å was used for all the systems to ensure the periodic images to be well separated. During the geometry optimizations, all the atoms were allowed to relax until the maximum magnitude of the force acting on the atoms is smaller than 0.03 eV/Å, and the total energy convergence criterion is set to 1×10^{-4} eV. The calculation of the Gibbs free energy of the intermediates followed the Nørskov method [S8].

The oxidation of hydrazine into nitrogen and hydrogen occurs in the following six consecutive elementary steps:

$$\text{(A) } * + N_2H_4 \rightarrow *N_2H_4 \quad \text{(S1)}$$

$$\text{(B) } *N_2H_4 \rightarrow *N_2H_3 + H^+ + e^- \quad \text{(S2)}$$

$$\text{(C) } *N_2H_3 \rightarrow *N_2H_2 + H^+ + e^- \quad \text{(S3)}$$

$$\text{(D) } *N_2H_2 \rightarrow *N_2H + H^+ + e^- \quad \text{(S4)}$$

$$\text{(E) } *N_2H \rightarrow *N_2 + H^+ + e^- \quad \text{(S5)}$$

$$\text{(F) } *N_2 \rightarrow * + N_2 \quad \text{(S6)}$$

The asterisk (*) represents the reaction surfaces. "$*N_2H_4$", "N_2H_3", "N_2H_2", "N_2H", and "$*N_2$" denote the models with the corresponding chemisorbed species residing in the reaction surfaces. Among these six elementary steps, steps (A) and (F) are the adsorption of N_2H_4 and desorption of N_2, respectively. The other four elementary steps involve the generation of one proton and one electron. Then, using the computational hydrogen electrode (pH = 0, p = 1 atm, T = 298 K), the Gibbs free energy of $H^+ + e^-$

was replaced implicitly with the Gibbs free energy of one-half an H_2 molecule. Thus the reaction Gibbs free energies can be calculated with Eqs:

$$\triangle G_A = \triangle G_{*N2H4} - \triangle G_{*} - \triangle G_{N2H4} \quad (S7)$$

$$\triangle G_B = \triangle G_{*N2H3} + 0.5\triangle G_{H2} - \triangle G_{*N2H4} - eU - kTIn10*pH \quad (S8)$$

$$\triangle G_C = \triangle G_{*N2H2} + 0.5\triangle G_{H2} - \triangle G*_{N2H3} - eU - kTIn10*pH \quad (S9)$$

$$\triangle G_D = \triangle G_{*N2H} + 0.5\triangle G_{H2} - \triangle G_{*N2H2} - eU - kTIn10*pH \quad (S10)$$

$$\triangle G_E = \triangle G_{*N2} + 0.5\triangle G_{H2} - \triangle G_{*N2H} - eU - kTIn10*pH \quad (S11)$$

$$\triangle G_F = \triangle G_{*} + G_{N2} - \triangle G_{*N2} \quad (S12)$$

U and the pH value in this work is set to zero. The adsorption or reaction Gibbs free energy is defined as $\triangle G = \triangle E + (ZPE - T\triangle S)$, where ΔE is the adsorption or reaction energy based on DFT calculations, ΔZPE is the zero-point energy (ZPE) correction, T is the temperature, and ΔS is the entropy change. For each system, its ZPE can be calculated by summing vibrational frequencies overall normal modes ν ($ZPE = 1/2\Sigma\hbar\nu$). The entropies of gas-phase H_2, N_2, and NH_2NH_2 are obtained from the NIST database[3] with the standard condition, and the adsorbed species were only taken vibrational entropy (S_v) into account, as shown in the following formula:

$$S_v = \Sigma i\ R\{hv_i/[k_BT*\exp(hv_i/k_BT) - k_BT] - In[1 - \exp(-hv_i/k_BT)]\} \quad (S13)$$

Among which $R = 8.314\ J\cdot mol^{-1}\cdot K^{-1}$, $T = 298.15$ K, $h = 6.63 \times 10^{-34}$ J·s, $k_B = 1.38 \times 10^{-23}\ J\cdot K^{-1}$, i is the frequency number, v_i is the vibrational frequency (unit is cm^{-1}).

Under the standard condition, the overall HER pathway includes two steps: first, adsorption of hydrogen on the catalytic site (*) from the initial state ($H^+ + e^- + *$), second, release the product hydrogen ($1/2H_2$). The total energies of $H^+ + e^-$ and $1/2H_2$ are equal. Therefore, the Gibbs free energy of the adsorption of the intermediate hydrogen on a catalyst (ΔG_H) is the key descriptor of the HER activity of the catalyst and is obtained by:

$$\Delta G_H = \Delta E_H + \Delta ZPE - T\Delta S$$

where ΔE_H, ΔZPE and ΔS are the adsorption energy, zero-point energy change and entropy change of H adsorption, respectively.

The kinetic energy barrier of the initial water dissociation step (ΔG_{H2O}) is applied as an activity descriptor for HER under alkaline condition, which can be calculated as follows:

$$\Delta G_{H2O} = G_{ts} - G_{ini}$$

where G_{ts} and G_{ini} are the free energy of the transient state and the initial state for water dissociation, respectively.

S2 Supplementary Figures and Tables

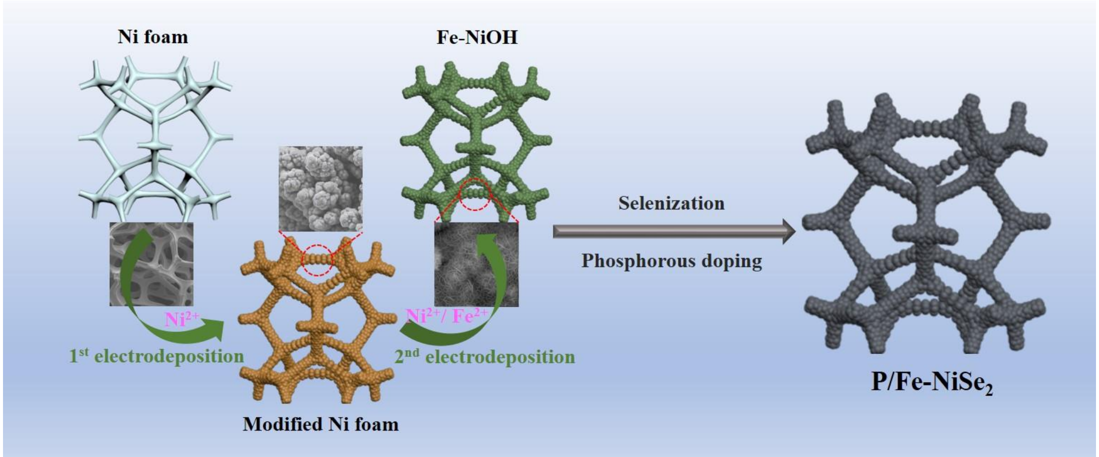

Scheme S1 Schematic illustration of the preparation of P/Fe-$NiSe_2$

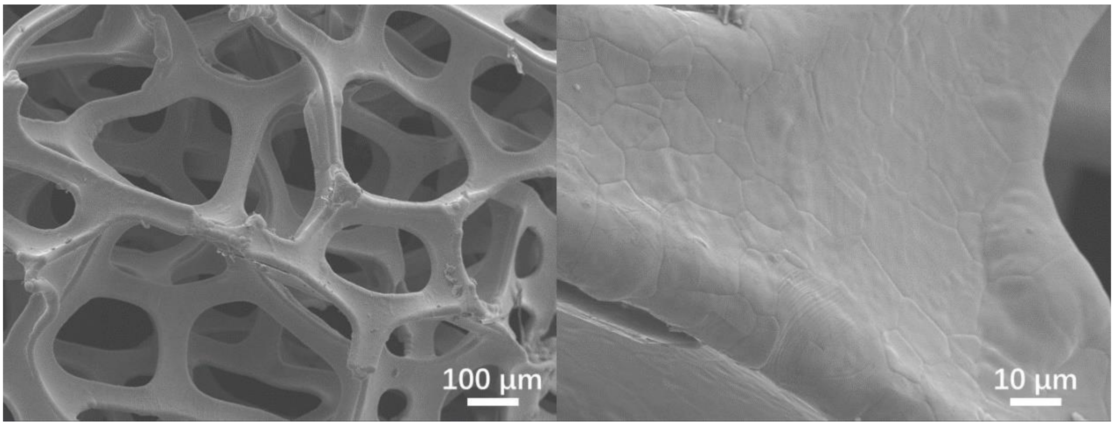

Fig. S1 SEM images of original Ni foam

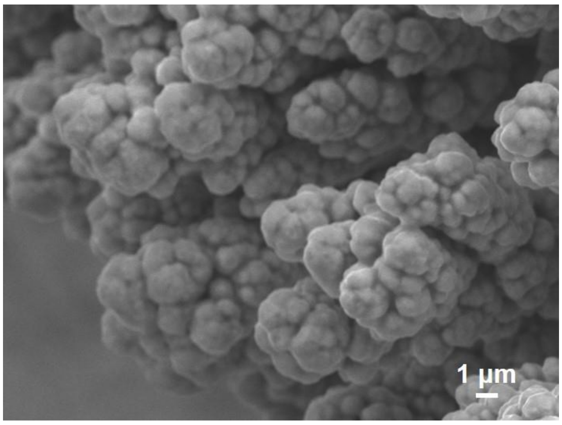

Fig. S2 High-magnification SEM image of Ni microsphere-modified Ni foam

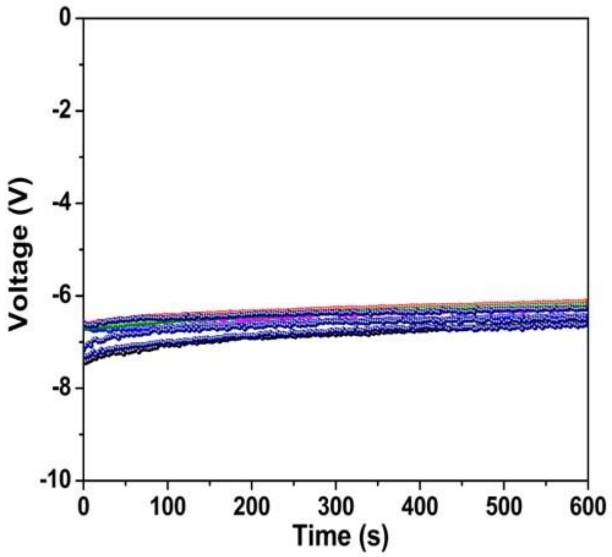

Fig. S3 Current densities in 1st electrodeposition

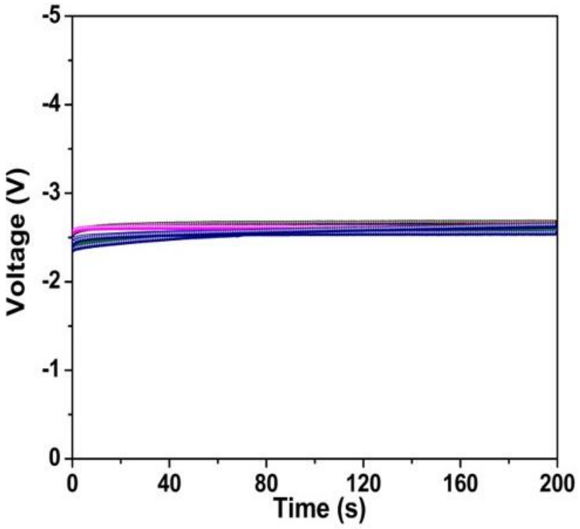

Fig. S4 Current densities in 2nd electrodeposition

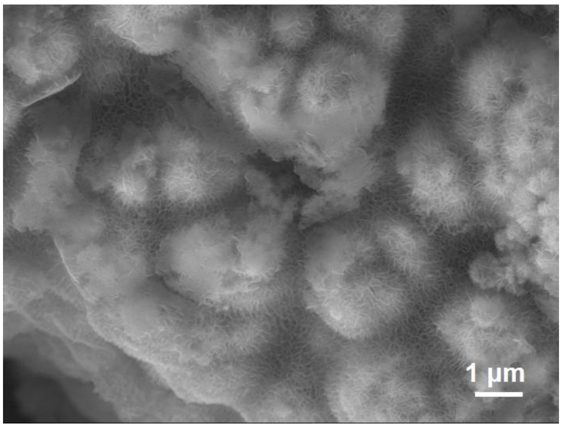

Fig. S5 High-magnification SEM image of Fe-NiOH

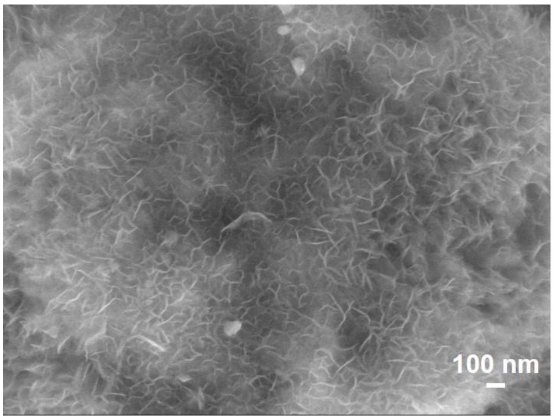

Fig. S6 High-magnification SEM image of Fe-$NiSe_2$

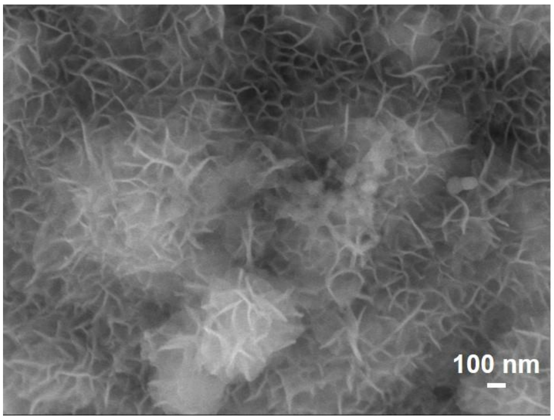

Fig. S7 High-magnification SEM image of $NiSe_2$

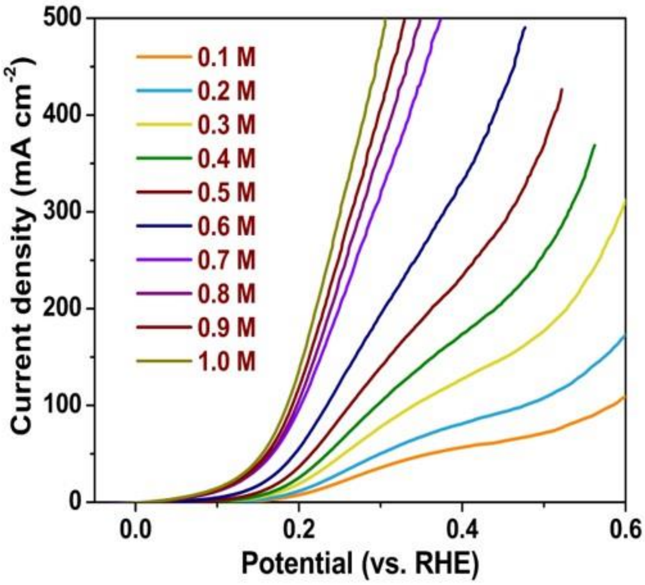

Fig. S8 LSV curves of P/Fe-$NiSe_2$ for HzOR in 1.0 M KOH with different concentration of N_2H_4

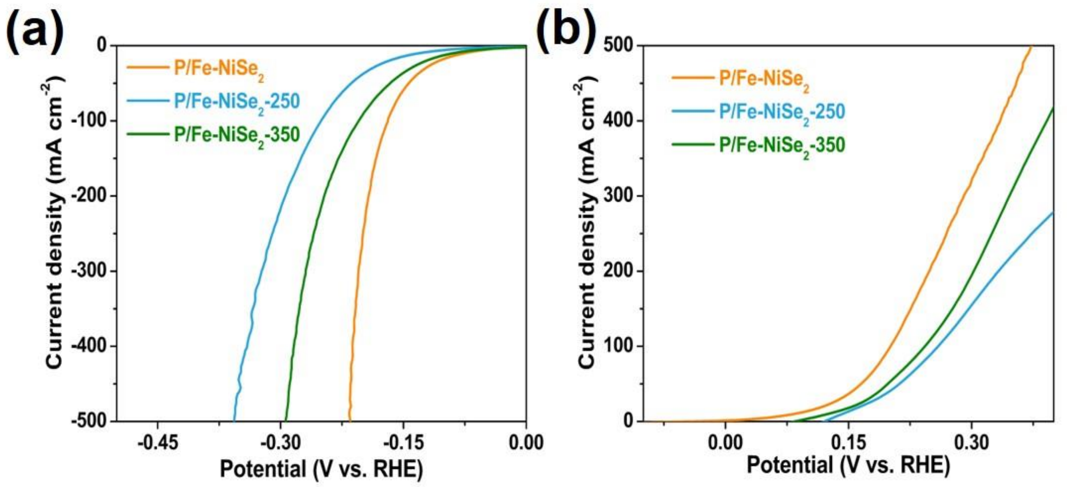

Fig. S9 LSV curves of P/Fe-NiSe₂, P/Fe-NiSe₂-250 and P/Fe-NiSe₂-350 for (**a**) HER and (**b**) HzOR

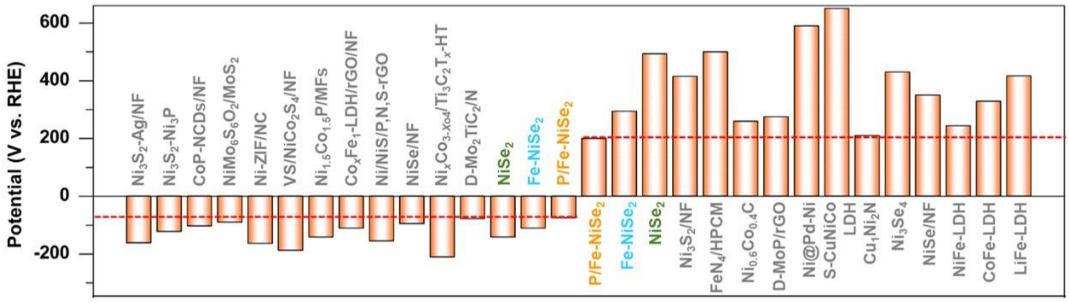

Fig. S10 Comparison of potentials for HER (10 mA cm^{-2}) and HzOR (100 mA cm^{-2}) of the synthesized electrocatalysts and some recently reported electrocatalysts

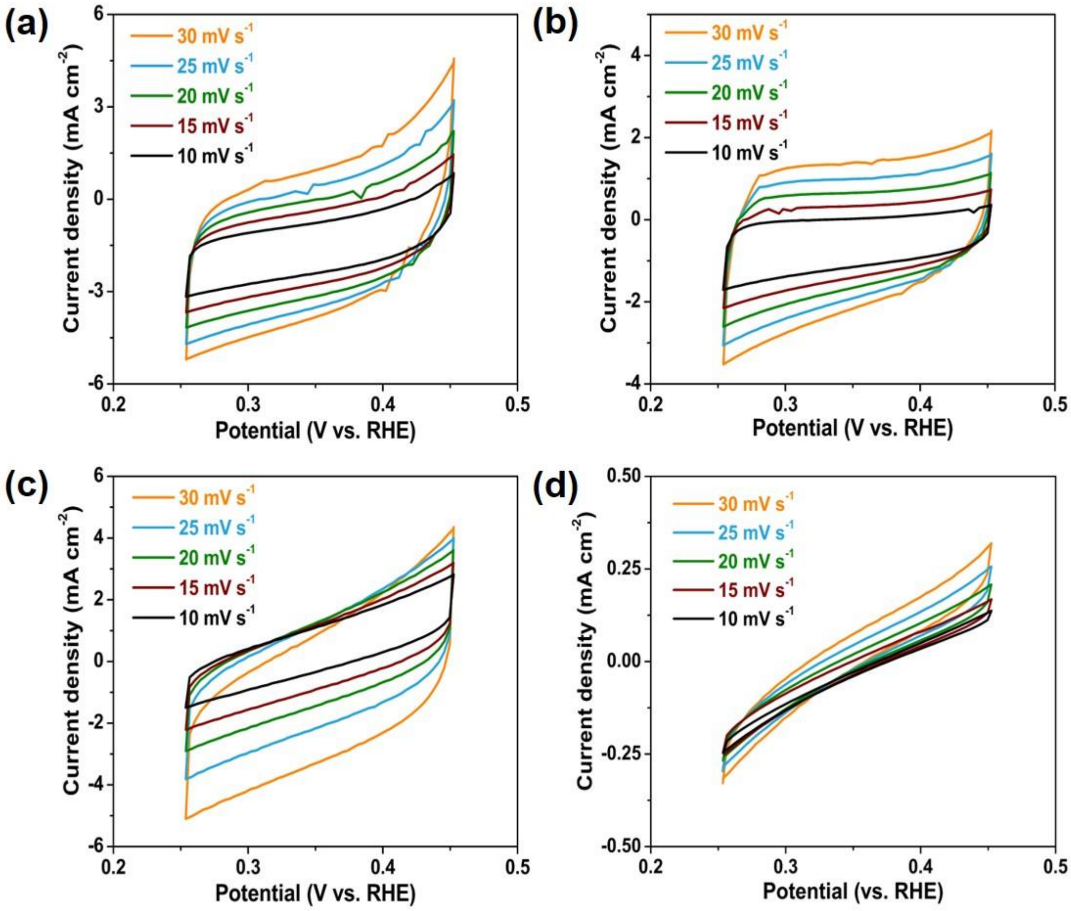

Fig. S11 Cyclic voltammograms of (**a**) P/Fe-NiSe$_2$, (**b**) Fe-NiSe$_2$, (**c**) NiSe$_2$ and (**d**) NF with different scan rates from 10 to 30 mV s^{-1}

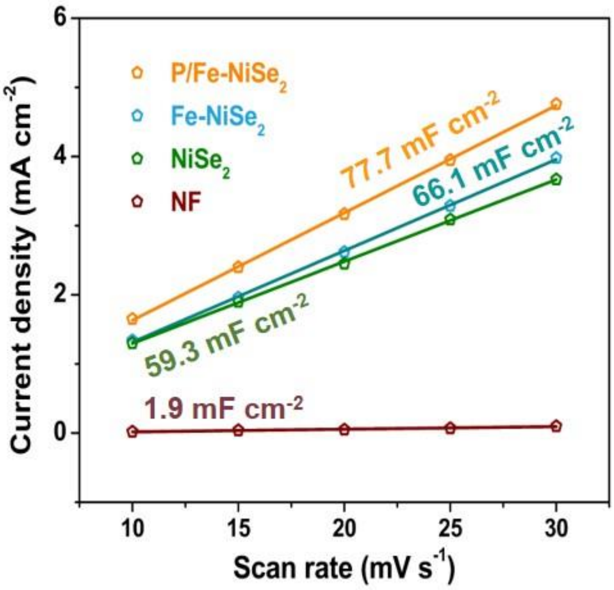

Fig. S12 C_{dl} conducted by plotting the current density difference against scan rate

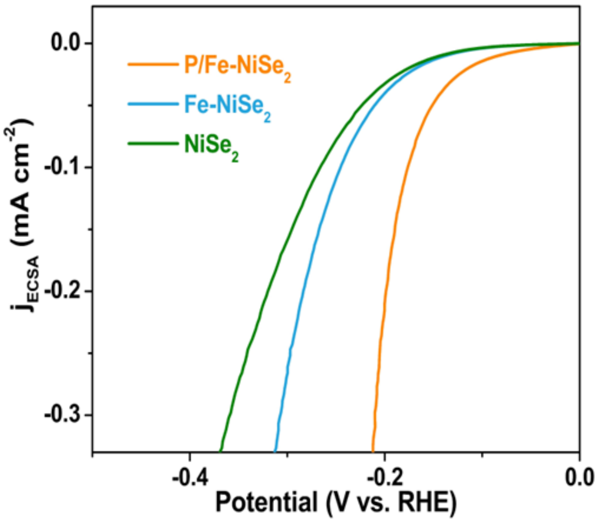

Fig. S13 HER Polarization curves with current densities normalized to the ECSA

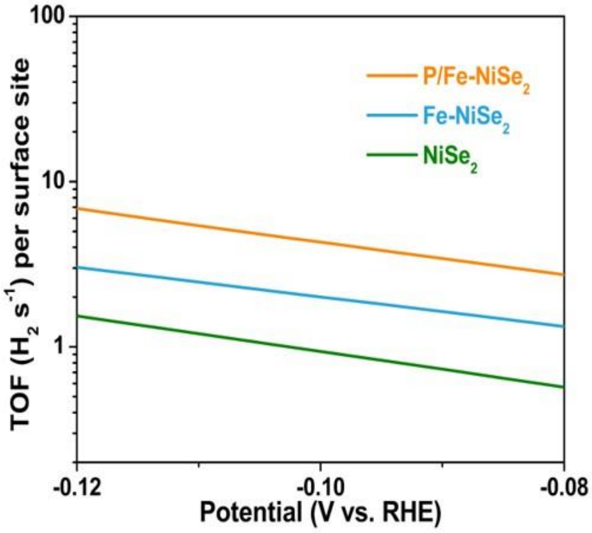

Fig. S14 Calculated H_2 TOFs

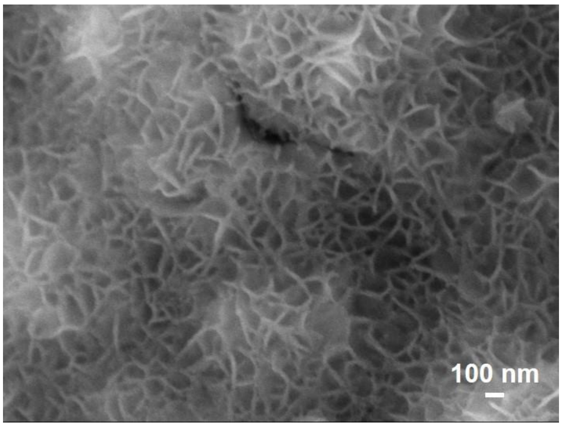

Fig. S15 High-magnification SEM image of post-HER P/Fe-$NiSe_2$

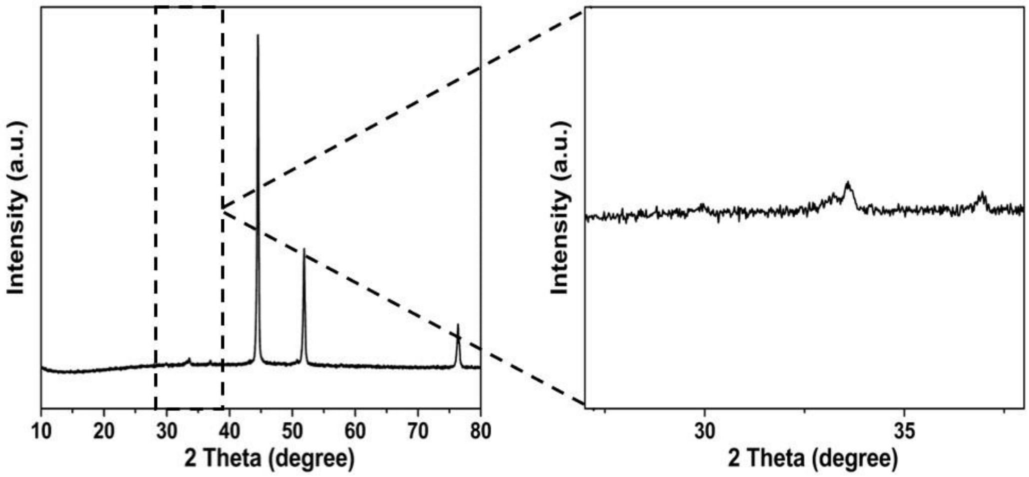

Fig. S16 XRD patterns of post-HER P/Fe-NiSe$_2$

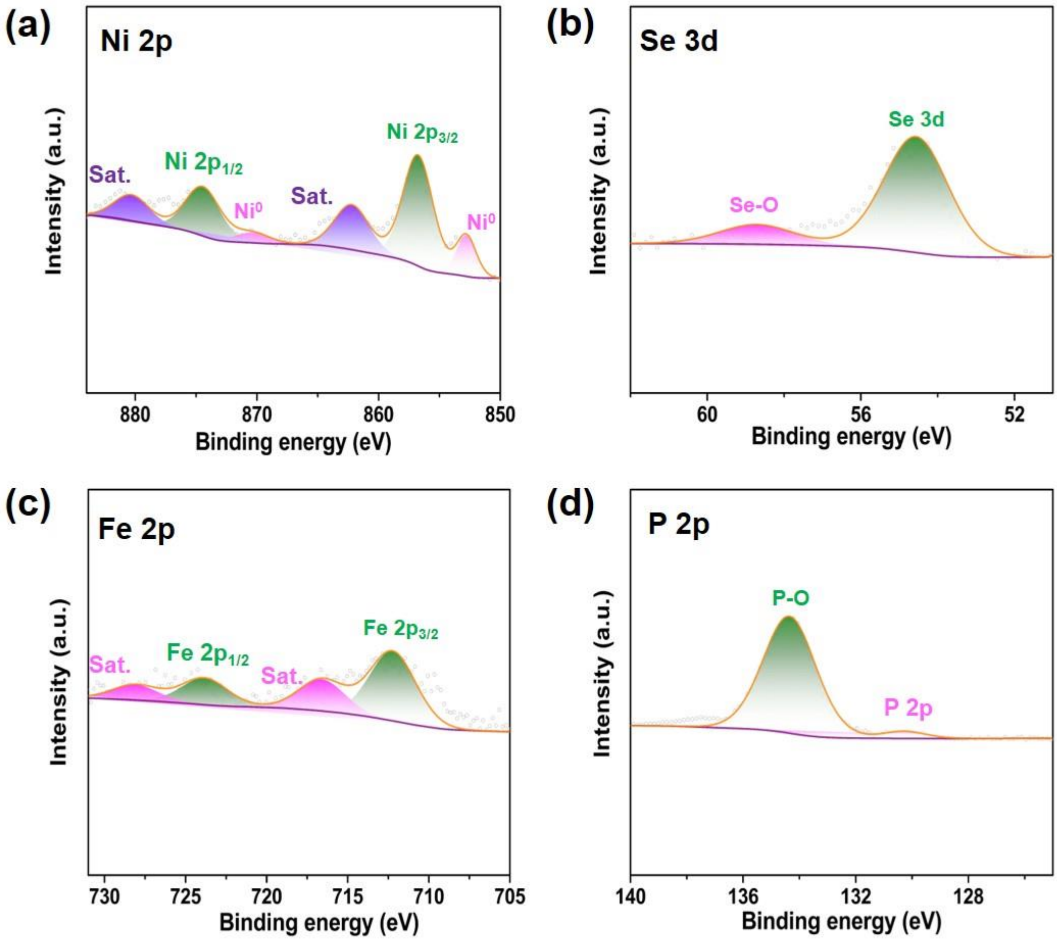

Fig. S17 XPS analysis of post-HER P/Fe-NiSe$_2$

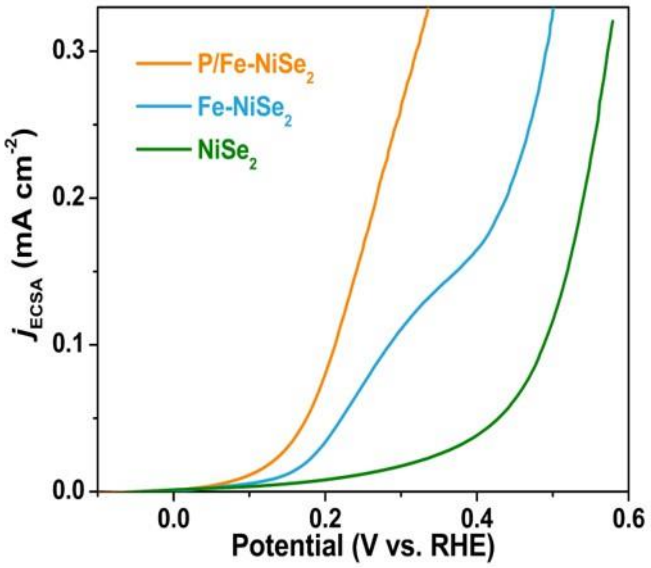

Fig. S18 HzOR polarization curves with current densities normalized to the ECSA

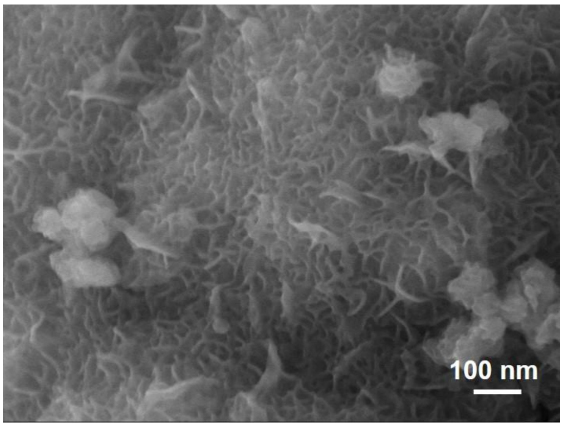

Fig. S19 High-magnification SEM image of post-HzOR P/Fe-$NiSe_2$

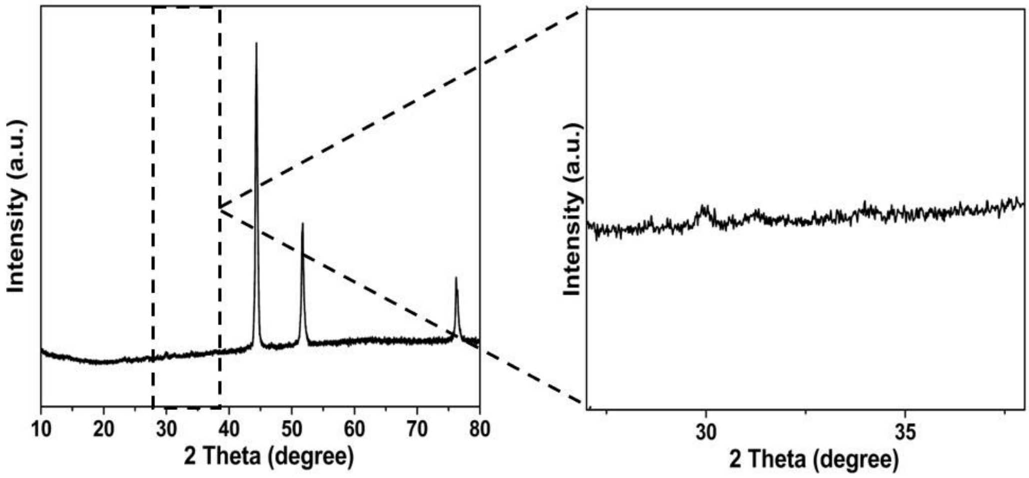

Fig. S20 XRD patterns of post-HzOR P/Fe-$NiSe_2$

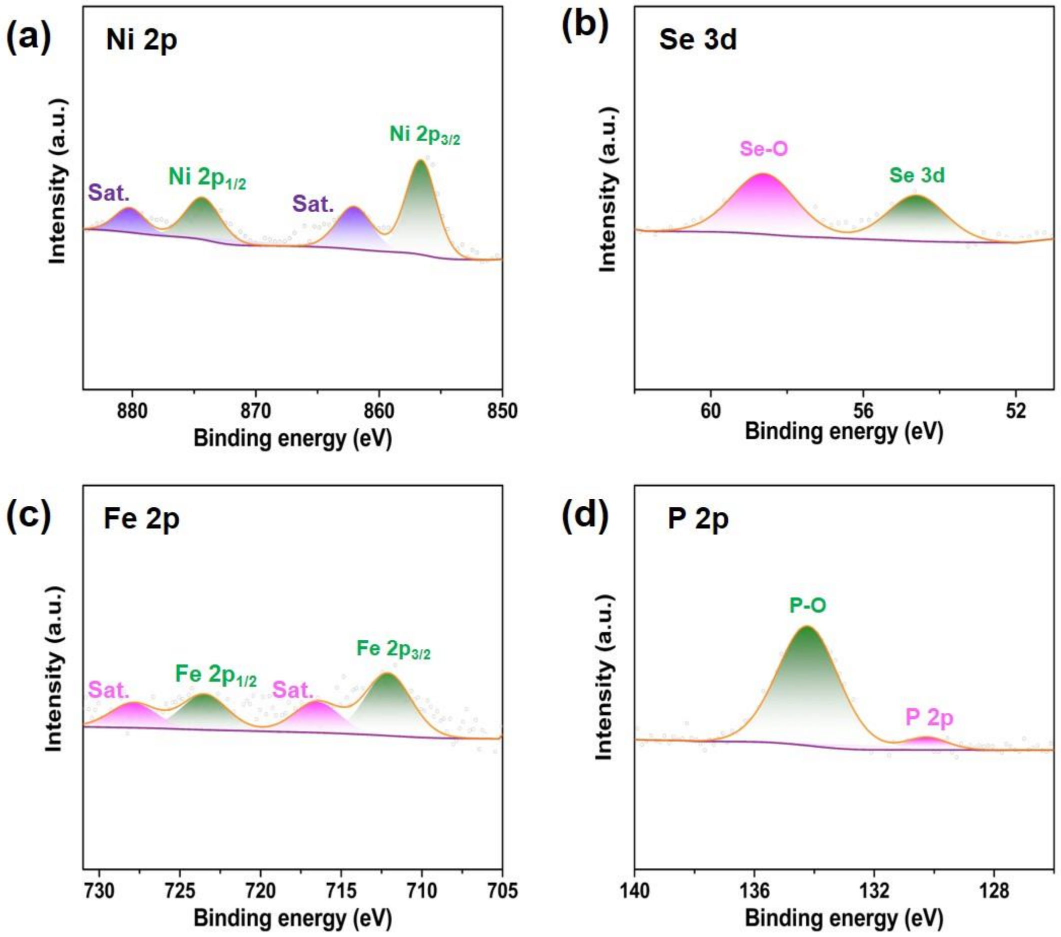

Fig. S21 XPS analysis of post-HzOR P/Fe-$NiSe_2$

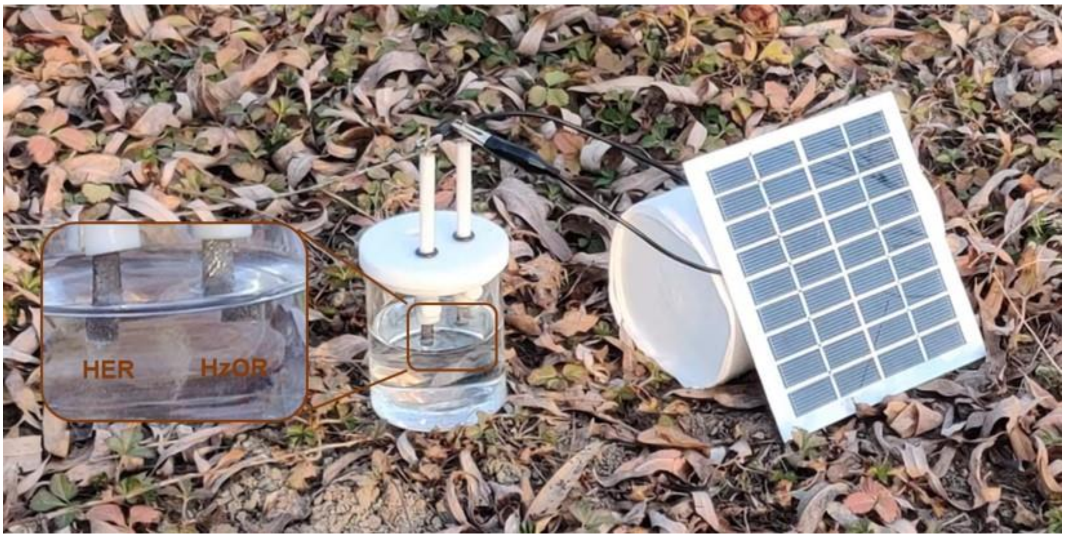

Fig. S22 Photo graph of a commercial solar panel powered OHzS device under sunlight. Inset: enlarged image of the electrode surface

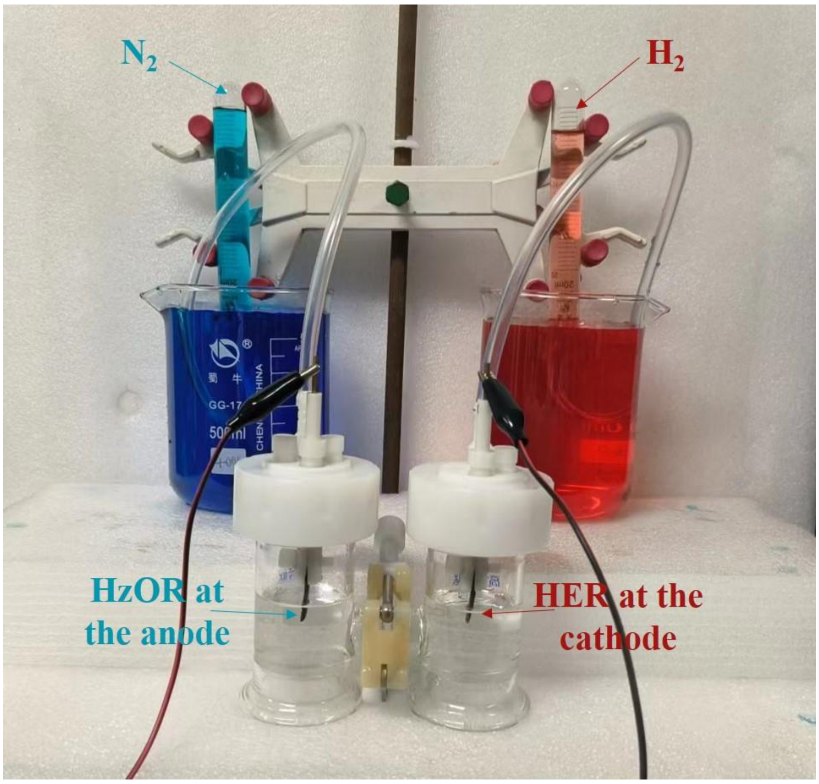

Fig. S23 Photograph of the gas measuring device for P,Fe-$NiSe_2$ in hydrazine-assisted water electrolysis with the H_2/N_2 ratio of about 2:1

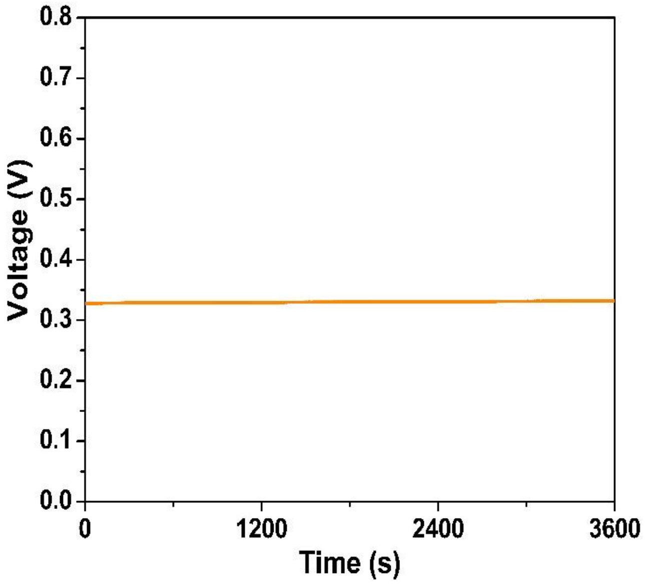

Fig. S24 Open circuit voltage for P,Fe-$NiSe_2$-assmbled Zn-Hz battery

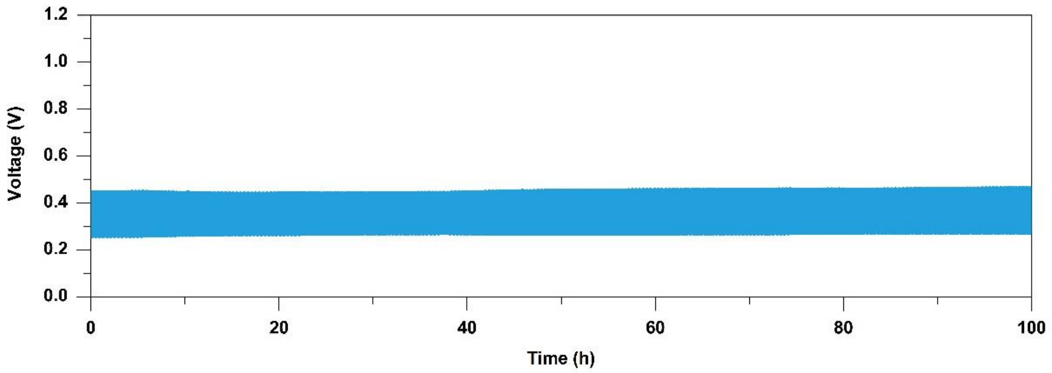

Fig. S25 Galvanostatic discharge-charge cycling curves for the P/Fe-$NiSe_2$-assembled Zn-Hz battery

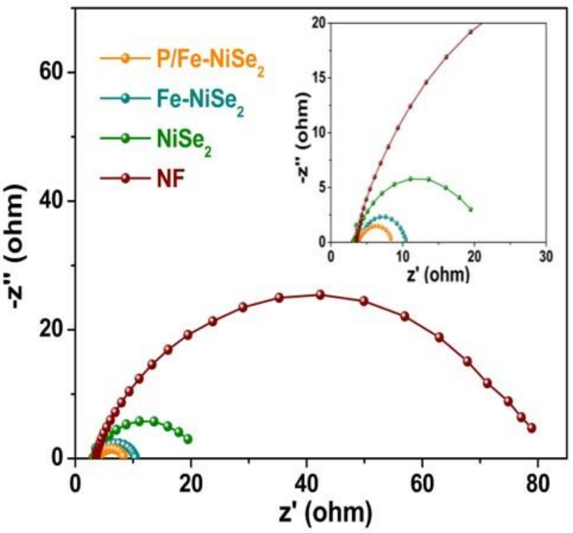

Fig. S26 Nyquist plots of collected at -0.1 V (vs. RHE). Inset: the enlarged view

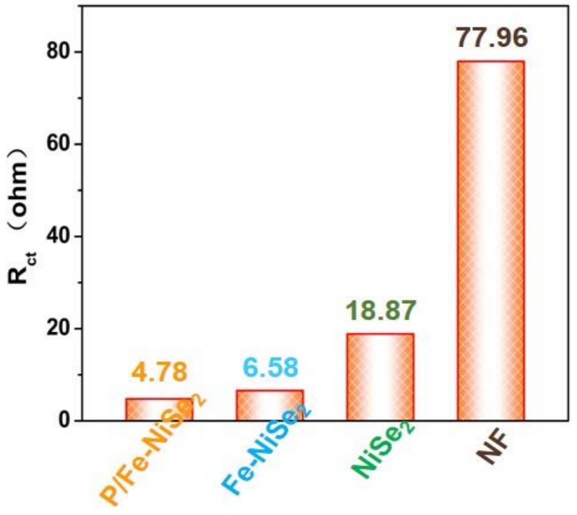

Fig. S27 R_{ct} of P/Fe-$NiSe_2$, Fe-$NiSe_2$, $NiSe_2$ and NF

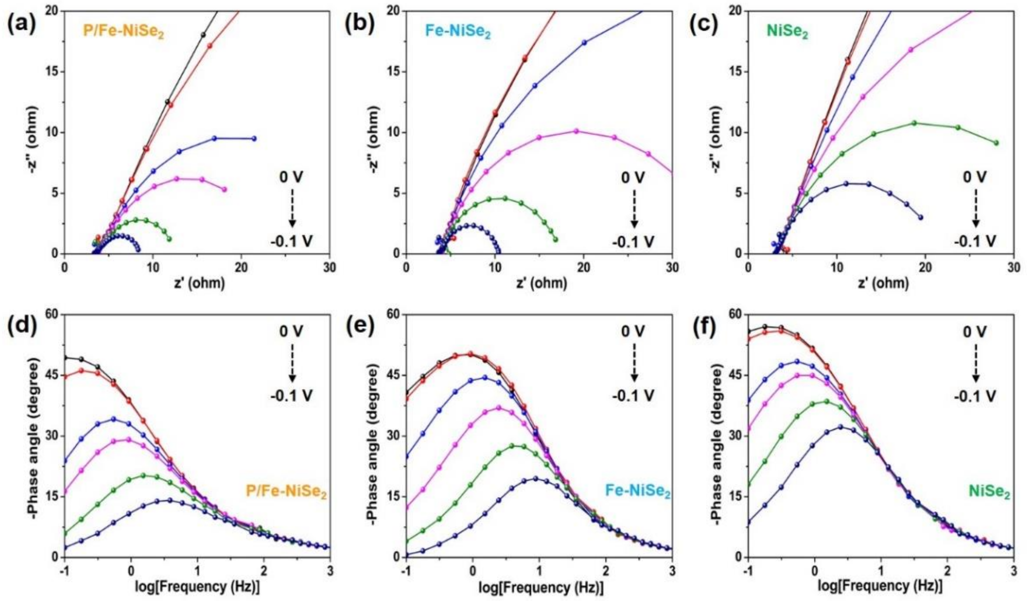

Fig. S28 (**a, b, c**) Nyquist and (**d, e, f**) Bode phase plots of P/Fe-NiSe$_2$, Fe-NiSe$_2$ and NiSe$_2$ at different potentials

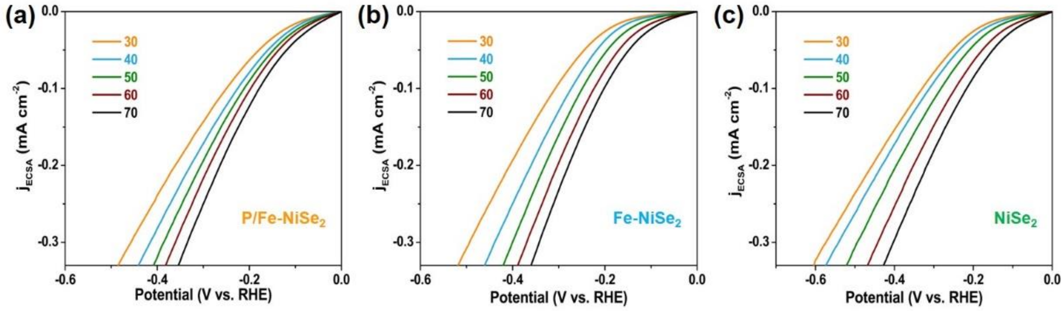

Fig. S29 Polarization curves without iR-corrected in the temperature range from 30 to 70 °C

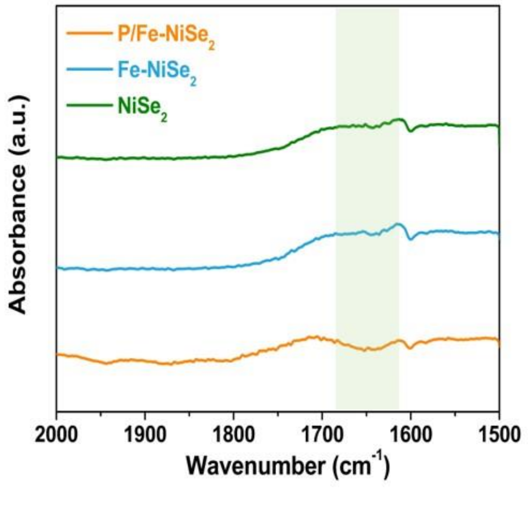

Fig. S30 Electrochemical in-situ FTIR spectroscopy on different electrocatalysts measured on -0.1 V (vs. RHE)

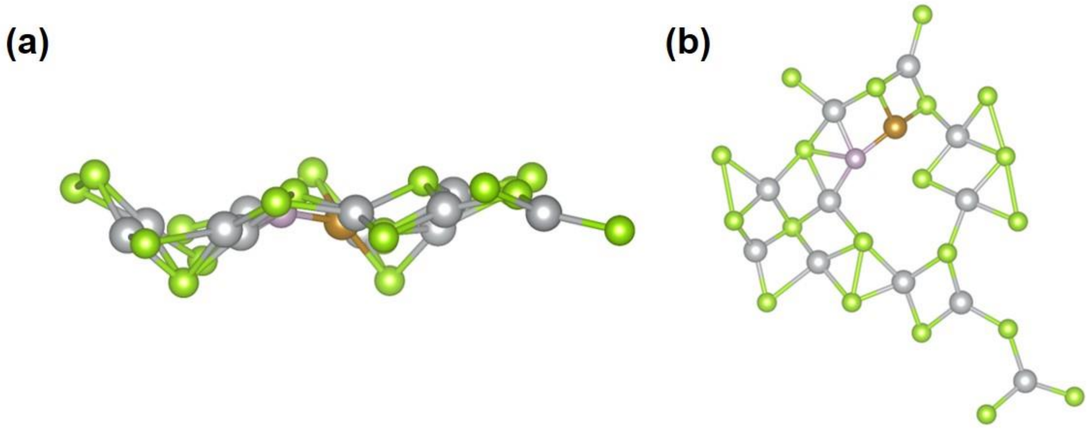

Fig. S31 (**a**) Top- and (**b**) side- view of atomic structure models for P,Fe-$NiSe_2$. The green, grey, brown and purple balls represent Se, Ni, Fe and P atoms, respectively

Table S1 Comparison of HER performance of P/Fe-$NiSe_2$ with other electrocatalysts

Catalyst	Electrolyte	Overpotential at 10 mA cm^{-2} (mV)	References
P/Fe-$NiSe_2$	1.0 M KOH	74	This work
Fe-$NiSe_2$	1.0 M KOH	110	This work
$NiSe_2$	1.0 M KOH	141	This work
D-Mo_2TiC_2/N	-	78	[S9]
$Ni_xCo_{3-x}O_4$/$Ti_3C_2T_x$-HT	1.0 M KOH	210	[S10]
NiSe/NF	1.0 M KOH	95	[S11]
Ni/NiS/P,N,S-rGO	1.0 M KOH	155	[S12]
Co_xFe_1-LDH/rGO/NF	1.0 M KOH	110	[S13]
$Ni_{1.5}Co_{1.5}P$/MFs	1.0 M KOH	141	[S14]
VS/$NiCo_2S_4$/NF	1.0 M KOH	187	[S15]
Ni-ZIF/NC	1.0 M KOH	163	[S16]
Ni-ZIF/NC	0.5 M H_2SO_4	177	[S16]
$NiMo_6S_6O_2$/MoS_2	1.0 M NaOH	90	[S17]
Fe-Co-O/Co@NC-mNS/NF	1.0 M KOH	112	[S18]
CoP-NCDs/NF	1.0 M KOH	103	[S19]
Ni_3S_2-Ni_3P	1.0 M KOH + 0.5 M urea	122	[S20]
Ni_3S_2-Ag/NF	1.0 M KOH	161	[S21]
Ni-Co-B	1.0 M KOH	145	[S22]
Ni_2P-Co_2P	1.0 M KOH	93	[S23]
Ni_2P-Co_2P	0.5 M H_2SO_4	172	[S23]
$Ni_{0.5}$@MoC_x/NC	0.5 M H_2SO_4	100	[S24]
Ni-Mo_2C@NPC	0.5 M H_2SO_4	144	[S25]
Ni-Mo_2C@NPC	1.0 M KOH	183	[S25]

Table S2 Comparison of HzOR performance of P/Fe-$NiSe_2$ with other electrocatalysts

Catalyst	Electrolyte	Potential at 100 mA cm^{-2} (mV vs. RHE)	References
P/Fe-$NiSe_2$	1.0 M KOH + 0.7 M N_2H_4	200	This work
Fe-$NiSe_2$	1.0 M KOH + 0.7 M N_2H_4	294	This work
$NiSe_2$	1.0 M KOH + 0.7 M N_2H_4	493	This work
Ni_3S_2/NF	1.0 M KOH + 0.2 M N_2H_4	415	[S26]
FeN_4/HPCM	1.0 M KOH + 0.1 M N_2H_4	500	[S27]
$Ni_{0.6}Co_{0.4}Se$	1.0 M KOH + 0.1 M N_2H_4	260	[S28]
D-MoP/rGO	1.0 M KOH + 0.5 M N_2H_4	275	[S29]
Ni@Pd-Ni	1.0 M KOH + 0.08 M N_2H_4	590	[S30]
S-CuNiCo LDH	1.0 M KOH + 0.02 M N_2H_4	650	[S31]
Cu_1Ni_2-N	1.0 M KOH + 0.5 M N_2H_4	210	[S32]
Ni_3Se_4	1.0 M KOH + 0.5 M N_2H_4	430	[S33]
NiSe/NF	1.0 M KOH + 0.5 M N_2H_4	350	[S11]
NiFe-LDH	1.0 M KOH + 2.0 M N_2H_4	244	[S34]
CoFe-LDH	1.0 M KOH + 2.0 M N_2H_4	329	[S34]
LiFe-LDH	1.0 M KOH + 2.0 M N_2H_4	417	[S34]

Table S3 Comparison of overall hydrazine splitting performance of P/Fe-$NiSe_2$ with other electrocatalysts

Catalyst	Electrolyte	Voltage at 10 or 100 mA cm^{-2} (mV)	References
P/Fe-$NiSe_2$	1.0 M KOH + 0.7 M N_2H_4	91 (10)	This work
P/Fe-$NiSe_2$	1.0 M KOH + 0.7 M N_2H_4	445 (100)	This work
Ni_3S_2/NF	1.0 M KOH + 0.2 M N_2H_4	867 (100)	[S26]
D-MoP/rGO	1.0 M KOH + 0.5 M N_2H_4	740 (100)	[S29]
Cu_1Ni_2-N	1.0 M KOH + 0.5 M N_2H_4	240 (10)	[S32]
Pd_1Co_1-CNFs	3.0 M KOH + 0.2 M N_2H_4	440 (10)	[S35]
Ni(Cu)@NiFeP/NM	1.0 M KOH + 0.5 M N_2H_4	491 (100)	[S36]
NiSe/NF	1.0 M KOH + 0.5 M N_2H_4	310 (10)<br>550 (100)	[S11]
NiOOH@CoCu CH	1.0 M KOH + 0.5 M N_2H_4	87 (10)<br>550 (100)	[S37]

Table S4 Gibbs energies for HzOR intermediates

Catalyst	*N_2H_4	*N_2H_3	*N_2H_2	*N_2H	*N_2
P/Fe-$NiSe_2$	-0.68	-0.75	-0.13	-0.17	-0.94
Fe-$NiSe_2$	-1.32	-1.93	-0.65	-0.76	-1.63

Supplementary References

[S1] Y. Duan, N. Dubouis, J. Huang, D.A. Dalla Corte, V. Pimenta, et al., Revealing the Impact of Electrolyte Composition for Co-Based Water Oxidation Catalysts by the Study of Reaction Kinetics Parameters, ACS Catal. **10**(7) 4160-4170 (2020). https://doi.org/10.1021/acscatal.0c00490

[S2] J. Huang, H. Sheng, R.D. Ross, J. Han, X. Wang, et al., Modifying redox properties and local bonding of Co_3O_4 by CeO_2 enhances oxygen evolution catalysis in acid, Nat. Commun. **12**(1) 3036 (2021). https://doi.org/10.1038/s41467-021-23390-8

[S3] G. Kresse, D. Joubert, From ultrasoft pseudopotentials to the projector augmented-wave method, Phys. Rev. B **59**(3) 1758-1775 (1999). https://doi.org/10.1103/PhysRevB.59.1758

[S4] G. Kresse, J. Hafner, Ab initio molecular dynamics for liquid metals, Phys. Rev. B **47**(1) 558-561 (1993). https://doi.org/10.1103/PhysRevB.47.558

[S5] G. Kresse, J. Furthmüller, Efficient iterative schemes for ab initio total-energy calculations using a plane-wave basis set, Phys. Rev. B **54**(16) 11169-11186 (1996). https://doi.org/10.1103/PhysRevB.54.11169

[S6] J.P. Perdew, K. Burke, M. Ernzerhof, Generalized Gradient Approximation Made Simple, Phys. Rev. Lett. **77**(18) 3865-3868 (1996). https://doi.org/10.1103/PhysRevLett.77.3865

[S7] H.J. Monkhorst, J.D. Pack, Special points for Brillouin-zone integrations, Phys. Rev. B **13**(12) 5188-5192 (1976). https://doi.org/10.1103/PhysRevB.13.5188

[S8] J.K. Nørskov, J. Rossmeisl, A. Logadottir, L. Lindqvist, J.R. Kitchin, et al., Origin of the Overpotential for Oxygen Reduction at a Fuel-Cell Cathode, J. Phys. Chem. B **108**(46) 17886-17892 (2004). https://doi.org/10.1021/jp047349j

[S9] Y. Zhu, G. Xu, W. Song, Y. Zhao, Z. He, et al., Anchoring single Ni atoms on defected 2D MXene nanosheets as an efficient electrocatalyst for enhanced hydrogen evolution reaction, Ceram. Int. **47**(21) 30005-30011 (2021). https://doi.org/10.1016/j.ceramint.2021.07.175

[S10] P. Xu, H. Wang, J. Liu, X. Feng, W. Ji, et al., High-Performance $Ni_xCo_{3-x}O_4/Ti_3C_2T_x$-HT Interfacial Nanohybrid for Electrochemical Overall Water Splitting, ACS Appl. Mater. Interfaces **13**(29) 34308-34319 (2021). https://doi.org/10.1021/acsami.1c08032

[S11] Y. Li, Y. Zhao, F.-M. Li, Z. Dang, P. Gao, Ultrathin NiSe Nanosheets on Ni Foam for Efficient and Durable Hydrazine-Assisted Electrolytic Hydrogen Production, ACS Appl. Mater. Interfaces **13**(29) 34457-34467 (2021).

https://doi.org/10.1021/acsami.1c09503

[S12] M.B.Z. Hegazy, M.R. Berber, Y. Yamauchi, A. Pakdel, R. Cao, et al., Synergistic Electrocatalytic Hydrogen Evolution in Ni/NiS Nanoparticles Wrapped in Multi-Heteroatom-Doped Reduced Graphene Oxide Nanosheets, ACS Appl. Mater. Interfaces **13**(29) 34043-34052 (2021). https://doi.org/10.1021/acsami.1c05888

[S13] J. Guo, Z. Wei, K. Wang, H. Zhang, Synergistic coupling of CoFe-layered double hydroxide nanosheet arrays with reduced graphene oxide modified Ni foam for highly efficient oxygen evolution reaction and hydrogen evolution reaction, Int. J. Hydrogen Energy **46**(54) 27529-27542 (2021). https://doi.org/10.1016/j.ijhydene.2021.06.013

[S14] T. Chen, M. Qian, X. Tong, W. Liao, Y. Fu, et al., Nanosheet self-assembled NiCoP microflowers as efficient bifunctional catalysts (HER and OER) in alkaline medium, Int. J. Hydrogen Energy **46**(58) 29889-29895 (2021). https://doi.org/10.1016/j.ijhydene.2021.06.121

[S15] Y. Han, S. Sun, J. Xu, X. Zhang, L. Wang, et al., Flocculent VS nanoparticle aggregate-modified $NiCo_2S_4$ nanograss arrays for electrocatalytic water splitting, Sustain. Energ. Fuels **5**(15) 3858-3866 (2021). https://doi.org/10.1039/D1SE00485A

[S16] Y. Zhang, S. Yun, M. Sun, X. Wang, L. Zhang, et al., Implanted metal-nitrogen active sites enhance the electrocatalytic activity of zeolitic imidazolate zinc framework-derived porous carbon for the hydrogen evolution reaction in acidic and alkaline media, J. Colloid Interface Sci. **604**(441-457 (2021). https://doi.org/10.1016/j.jcis.2021.06.152

[S17] H. Cheng, Y. Diao, Q. Liu, L. Wei, X. Li, et al., Di-nuclear metal synergistic catalysis: $Ni_2Mo_6S_6O_2/MoS_2$ two-dimensional nanosheets for hydrogen evolution reaction, Chem. Eng. J. **428** 131084 (2022). https://doi.org/10.1016/j.cej.2021.131084

[S18] T.I. Singh, G. Rajeshkhanna, U.N. Pan, T. Kshetri, H. Lin, et al., Alkaline Water Splitting Enhancement by MOF-Derived Fe–Co–Oxide/Co@NC-mNS Heterostructure: Boosting OER and HER through Defect Engineering and In Situ Oxidation, Small **17**(29) 2101312 (2021). https://doi.org/10.1002/smll.202101312

[S19] H. Liu, Z. Liu, Y. Wang, J. Zhang, Z. Yang, et al., Carbon dots-oriented synthesis of fungus-like CoP microspheres as a bifunctional electrocatalyst for efficient overall water splitting, Carbon **182**(327-334 (2021). https://doi.org/10.1016/j.carbon.2021.06.029

[S20] J. Liu, Y. Wang, Y. Liao, C. Wu, Y. Yan, et al., Heterostructured Ni_3S_2–Ni_3P/NF as a Bifunctional Catalyst for Overall Urea–Water Electrolysis for Hydrogen Generation, ACS Appl. Mater. Interfaces **13**(23) 26948-26959

(2021). https://doi.org/10.1021/acsami.1c04325

[S21] H.-J. Liu, W.-L. Yu, M.-X. Li, S.-Y. Dou, F.-L. Wang, et al., The rational design of Ni_3S_2 nanosheets–Ag nanorods on Ni foam with improved hydrogen adsorption sites for the hydrogen evolution reaction, Sustain. Energ. Fuels **5**(13) 3428-3435 (2021). https://doi.org/10.1039/D1SE00702E

[S22] A. Jokar, A. Toghraei, M. Maleki, G. Barati Darband, Facile electrochemical synthesis of Ni-Co-B film on Cu sheet for dual-electrocatalysis of hydrogen and oxygen evolution reactions, Electrochim. Acta **389** 138691 (2021). https://doi.org/10.1016/j.electacta.2021.138691

[S23] Q. Cao, S. Hao, Y. Wu, K. Pei, W. You, et al., Interfacial charge redistribution in interconnected network of Ni_2P–Co_2P boosting electrocatalytic hydrogen evolution in both acidic and alkaline conditions, Chem. Eng. J. **424** 130444 (2021). https://doi.org/10.1016/j.cej.2021.130444

[S24] C. Liu, L. Sun, L. Luo, W. Wang, H. Dong, et al., Integration of Ni Doping and a Mo_2C/MoC Heterojunction for Hydrogen Evolution in Acidic and Alkaline Conditions, ACS Appl. Mater. Interfaces **13**(19) 22646-22654 (2021). https://doi.org/10.1021/acsami.1c04989

[S25] Y. Lu, C. Yue, Y. Li, W. Bao, X. Guo, et al., Atomically dispersed Ni on Mo_2C embedded in N, P co-doped carbon derived from polyoxometalate supramolecule for high-efficiency hydrogen evolution electrocatalysis, Appl. Catal. B-Environ. **296** 120336 (2021). https://doi.org/10.1016/j.apcatb.2021.120336

[S26] G. Liu, Z. Sun, X. Zhang, H. Wang, G. Wang, et al., Vapor-phase hydrothermal transformation of a nanosheet array structure $Ni(OH)_2$ into ultrathin Ni_3S_2 nanosheets on nickel foam for high-efficiency overall water splitting, J. Mater. Chem. A **6**(39) 19201-19209 (2018). https://doi.org/10.1039/C8TA07162D

[S27] Y.-C. Wang, L.-Y. Wan, P.-X. Cui, L. Tong, Y.-Q. Ke, et al., Porous Carbon Membrane-Supported Atomically Dispersed Pyrrole-Type Fe-N_4 as Active Sites for Electrochemical Hydrazine Oxidation Reaction, Small **16**(31) 2002203 (2020). https://doi.org/10.1002/smll.202002203

[S28] Z. Feng, E. Wang, S. Huang, J. Liu, A bifunctional nanoporous Ni–Co–Se electrocatalyst with a superaerophobic surface for water and hydrazine oxidation, Nanoscale **12**(7) 4426-4434 (2020). https://doi.org/10.1039/C9NR09959J

[S29] Y. Gao, Q. Wang, T. He, J.-Y. Zhang, H. Sun, et al., Defective crystalline molybdenum phosphides as bifunctional catalysts for hydrogen evolution and hydrazine oxidation reactions during water splitting, Inorg. Chem. Front. **6**(10) 2686-2695 (2019). https://doi.org/10.1039/C9QI01005J

[S30] M. Du, H. Sun, J. Li, X. Ye, F. Yue, et al., Integrative Ni@Pd-Ni Alloy

Nanowire Array Electrocatalysts Boost Hydrazine Oxidation Kinetics, ChemElectroChem **6**(22) 5581-5587 (2019). https://doi.org/10.1002/celc.201901303

[S31] W. Liu, J. Xie, Y. Guo, S. Lou, L. Gao, et al., Sulfurization-induced edge amorphization in copper–nickel–cobalt layered double hydroxide nanosheets promoting hydrazine electro-oxidation, J. Mater. Chem. A **7**(42) 24437-24444 (2019). https://doi.org/10.1039/C9TA07857F

[S32] Z. Wang, L. Xu, F. Huang, L. Qu, J. Li, et al., Copper–Nickel Nitride Nanosheets as Efficient Bifunctional Catalysts for Hydrazine-Assisted Electrolytic Hydrogen Production, Adv. Energy Mater. **9**(21) 1900390 (2019). https://doi.org/10.1002/aenm.201900390

[S33] J.-Y. Zhang, X. Tian, T. He, S. Zaman, M. Miao, et al., In situ formation of Ni_3Se_4 nanorod arrays as versatile electrocatalysts for electrochemical oxidation reactions in hybrid water electrolysis, J. Mater. Chem. A **6**(32) 15653-15658 (2018). https://doi.org/10.1039/C8TA06361C

[S34] Z. Li, M. Shao, H. An, Z. Wang, S. Xu, et al., Fast electrosynthesis of Fe-containing layered double hydroxide arrays toward highly efficient electrocatalytic oxidation reactions, Chem. Sci. **6**(11) 6624-6631 (2015). https://doi.org/10.1039/C5SC02417J

[S35] Y. Ao, S. Chen, C. Wang, X. Lu, Palladium cobalt alloy encapsulated in carbon nanofibers as bifunctional electrocatalyst for high-efficiency overall hydrazine splitting, J. Colloid Interface Sci. **601** 495-504 (2021). https://doi.org/10.1016/j.jcis.2021.05.119

[S36] Q. Sun, M. Zhou, Y. Shen, L. Wang, Y. Ma, et al., Hierarchical nanoporous Ni(Cu) alloy anchored on amorphous NiFeP as efficient bifunctional electrocatalysts for hydrogen evolution and hydrazine oxidation, J. Catal. **373** 180-189 (2019). https://doi.org/10.1016/j.jcat.2019.03.039

[S37] B. Li, K. Wang, J. Ren, P. Qu, NiOOH@Cobalt copper carbonate hydroxide nanorods as bifunctional electrocatalysts for highly efficient water and hydrazine oxidation, New J. Chem. **46**(16) 7615-7625 (2022). https://doi.org/10.1039/D2NJ00518B
